# Combined Targeted Analysis of Metabolites and Proteins in Tear Fluid With Regard to Clinical Applications

**DOI:** 10.1167/tvst.7.6.22

**Published:** 2018-12-06

**Authors:** Sascha Dammeier, Peter Martus, Franziska Klose, Michael Seid, Dario Bosch, Janina D‘Alvise, Focke Ziemssen, Spyridon Dimopoulos, Marius Ueffing

**Affiliations:** 1 Institute for Ophthalmic Research, Core Facility for Medical Bioanalytics, University of Tübingen, Tübingen, Germany; 2 Clinical Epidemiology and Applied Biometry, University Hospital Tübingen, Tübingen, Germany; 3 Centre of Ophthalmology, University Eye Hospital Tübingen, Tübingen, Germany

**Keywords:** tear fluid, mass spectrometry, metabolomics, proteomics, eye

## Abstract

**Purpose:**

To establish a robust workflow for combined mass spectrometry–based analysis of metabolites and proteins in tear fluid with regard to clinical applicability.

**Methods:**

Tear fluid was taken from 12 healthy volunteers at different time points using specially designed Schirmer strips. Following the liquid extraction of metabolites from standardized punches, the remaining material was processed for bottom-up proteomics. Targeted metabolite profiling was performed adapting a metabolomics kit, which targets 188 metabolites from four different analyte classes. Proteomics was performed of the identical samples targeting 15 tear proteins relevant to ocular health.

**Results:**

Sixty metabolites could be consistently determined in all tear samples (98 metabolites were detectable in average) covering acylcarnitines, amino acids, biogenic amines, and glycerophospholipids. Following normalization, the majority of metabolites exhibited intraindividual variances of less than 20%, both regarding different times of sampling, and the individual eye. The targeted analysis of tear proteins revealed a mean intraindividual variation of 23% for the three most abundant proteins. Even extreme differences in tear secretion rates resulted in interindividual variability below 30% for 65 metabolites and two proteins.

**Conclusions:**

The newly established workflow can be used for combined targeted detection of metabolites and proteins in one punch of a Schirmer strip in a clinical setting.

**Translational Relevance:**

Our data about intra- and interindividual as well as intereye variation provide a valuable basis for the design of clinical studies, and for the applicability of multiplexed “omics” to well accessible tear fluid with regard to future routine use.

## Introduction

The tear fluid forms a film that covers the cornea and conjunctiva of the eye. It fulfills multiple tasks with regard to the functionality and integrity of the visual system. Among others, tear fluid protects epithelial cells against dehydration, removes particles and waste products, and provides nutrients as well as an antimicrobial defense system.^1^ It has been known for a long time that the excretion and constitution of tear fluid are affected by ocular diseases, for example in patients suffering from dry-eye syndrome.^2^ Thereby, the tear secretion rate is significantly reduced, and the osmolarity and hence the concentrations of molecular components are altered. Therefore, the composition of tear fluid is reflective of ocular homoeostasis and health. From a medical perspective, tear fluid has also been seen as a gateway for pharmaceutical intervention into the human eye. The classical examples are eye drops that have been formulated to treat infections, glaucoma, and inflammatory disorders.^3^

With respect to laboratory analytics, tear fluid represents an easily accessible sample material that has the potential to become a matrix for diagnostic analyses, both for eye-related diseases and systemic disorders.^4^ A prominent example of being a relevant body fluid for systemic diseases is that measuring the glucose concentration in tear fluid can serve as a less invasive surrogate for the determination of glucose levels in the blood (e.g., for monitoring diabetes).^5,6^ Apart from the detection of single markers like glucose or cytokines, the determination of multiplexed biomarkers will become even more relevant for diagnostics in the future.^7^ Complex diseases might also induce complex physiological changes that could primarily be detected by a combination of metabolomics, lipidomics, and proteomics.^4^

However, major practical hurdles regarding tear collection and preanalytics have not yet been fully overcome. Today, the Schirmer tear test is the most widely used technique to determine the production rate of tear fluid. In addition, it has been used “off label” to collect tear samples to perform special chemical analytics.^8^ An alternative method to collect tear fluid is sampling via glass capillaries, which is tedious and challenging. Elevated discomfort for the donors has reported to distort the secretion rates. Hence, sample collection using paper strips would be the preferred technique for routine application, and also the most economic. There have also been multiple approaches to use Schirmer strips for the collection of tear fluid to perform either proteomic or metabolomic research. By applying proteomics to tear proteins that have been eluted from Schirmer strips, biomarker signatures have been reported for dry-eye syndrome,^9,10^ diabetic retinopathy,^11,12^ keratoconjunctivitis,^13^ Sjogren's syndrome,^14^ multiple sclerosis,^15^ and primary open-angle glaucoma^16^; this just names a selection of studies that have primarily used mass spectrometry–based proteomics. Concerning metabolite analysis using tear fluid, biomarker studies have focused on dry-eye syndrome,^17–19^ Meibomian gland dysfunction,^19^ diabetes mellitus,^20^ aging,^8^ keratoconus,^21^ and ocular surface diseases.^22^

As indicated by the biomarker studies mentioned above, it would be highly desirable to perform multiplexed analyses from tear fluid if the application should become a real improvement for future disease management. In this regard, some studies have been reported both for targeted protein determination^23–25^ and for metabolomics.^26^ However, it is quite striking—to the best of our knowledge—so far, none of the studies combined metabolomics and proteomic analysis of the identical tear samples. That is most desirable, as the amount of sample in one Schirmer strip is limited, and the repetitive collection using strips is rather impractical. Moreover, because metabolites, lipids, and proteins together reflect the physiology and pathology of the eye and of systemic diseases more accurately,^27^ in this work we describe a workflow to measure both metabolites and proteins quantitatively in the identical tear fluid sample collected by specially designed Schirmer strips. To evaluate the method in the context of its potential clinical application, we also performed an assessment of intraindividual variability, intereye correlations, and interindividual variation of housekeeping proteins and metabolites on the basis of a pilot study with healthy subjects.

## Methods

### Materials and Chemicals

All solvents and water were purchased from Merck (Darmstadt, Germany) as HPLC-grade purity. All chemicals were purchased from Sigma Aldrich (Taufkirchen, Germany) unless stated otherwise. High performance liquid chromatography (HPLC) columns were obtained from Thermo Fisher Scientific (Dreieich, Germany) and from Waters (Eschborn, Germany) for proteomic and metabolomics analysis, respectively. The AbsoluteIDQ p180 kit for targeted and quantitative metabolome analysis was purchased from Biocrates Life Sciences (Innsbruck, Austria). The peptide standards DGAGDVAFIR, WESGYNTR, STDYGIFQINSR, GLSTESILIPR, FYTIEILKVE, AQAELENVSGALNEAESK, ELGEYGFHEYTEVK, LLEDMVEK, TINSDISIPEYK, SILLTEQALAK, SASDLTWDNLK, NFPSPVDAAFR, HHPALSPIAR, GNPTVEVDLFTSK, FPSVSLQEASSFFQR, LNSPLSLPFVPGR, and GIVDQSQQAYQEAFEISK were synthesized by JPT Peptide Technologies (Berlin, Germany). Polypropylene microtiter plates were purchased from VWR (Bruchsal, Germany). Whatmann filter paper grade 903 for the production of custom-made Schirmer-like test strips was obtained from GE Healthcare (Dassel, Germany). On the one hand, this material was certified as a medical product, but on the other hand it is known to be highly compatible with mass spectrometric sample preparation as it has been validated for metabolomics in the context of newborn screening. Schirmer strips were cut out of the plain 903 sheet using a custom-made punching device (35-mm length; 5-mm width) obtaining planar strips with one rounded tip. At the latter tip, a 90° fold was introduced at a distance of 5 mm from the end.

### Study Design and Tear Collection

Human tear fluid was collected from 12 healthy volunteers (6 female and 6 male volunteers, average age of 30.7 ± 5.4 years) who had no recent history of ocular disease. Written consent was obtained from all participants prior to study enrollment. The study followed the tenets of the Declaration of Helsinki and was approved by the ethics committee of the medical faculty of the Eberhard-Karls-University Tuebingen (ClinTrials.gov, registration number NCT03389282). Patients had a routine ophthalmic examination including best-corrected visual acuity (BCVA) using Early Treatment of Diabetic Retinopathy Study (ETDRS) charts, slit-lamp biomicroscopy, and indirect ophthalmoscopy at baseline. Tear breakup time (TBUT) test was used to assess evaporative dry-eye disease. To measure TBUT, fluorescein was instilled into the patient's tear film and the patient was asked not to blink while the tear film was observed under a broad beam of cobalt blue illumination. Fluorescein was also used to rule out any pathologies of the conjunctiva or the cornea, such as punctate epithelial erosions (PEE). To take most of common environmental and nutritional influences into account, the samples were taken during three independent visits (i.e., on 3 different days at different time points). Thus, on each collection day, a different time point was chosen, namely in the morning, at noon, or in the early afternoon. However, time elapsed between the last meal and sample collection was not monitored. For pairwise comparison, tears were collected both from the left and the right eye. Tear samples were collected by special Schirmer-like filter papers without using local anesthesia. The strips were inserted for 5 minutes in the lower eyelid in the standard fashion in both eyes consecutively. The study protocol required that in the case of subjects who exhibited a very high tear secretion rate, the collection was stopped when the strip was filled with tear fluid, and the time was documented. After collection, all strips were air dried at room temperature until completely dry. Then the strips were stored in paper envelopes at −20°C until analysis.

### Sample Preparation

**Figure 1:**
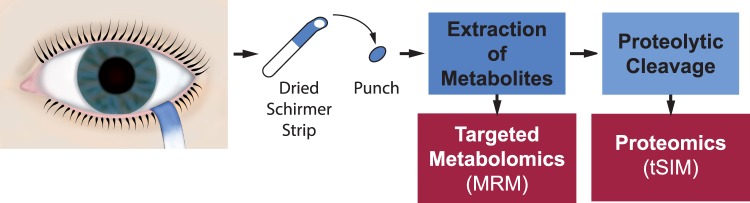
Sample preparation workflow following tear fluid donation using Schirmer strips. At first, a distinct punch of the dried Schirmer strip was extracted to receive a metabolite fraction that was used for mass-spectrometric metabolome analysis based on MRM. Second, the identical punch was processed using limited proteolysis, recovery of the resulting peptides and proteomic analysis with t-SIM mass spectrometry.

An overview of the sample preparation workflow is given in Figure 1. Punches of 4 mm in diameter were cut out of the front end of each Schirmer-like strip using a custom-made punching tool. Then, the punches were directly transferred into the wells of the 96-well plate of the metabolomics kit.

### Metabolite Extraction

To perform a highly standardized, well-reproducible metabolite extraction and analysis, the AbsoluteIDQ p180 kit was used. The kit plate containing the Schirmer punches was processed according to the manufacturer's instructions. Finally, approximately 300 μL of a methanol-based metabolite extract was obtained for each tear sample. The remaining filter plate of the kit containing the one-time extracted punches was removed and dried under vacuum for 30 minutes. The punches were transferred to a 96-well plate either for direct protein digestion or for storage at −20°C until further processing.

### Protein Digestion

The pre-extracted punches and, for comparison, Schirmer punches that had not undergone metabolomics extraction were incubated in 100 μL of 50 mM ammonium bicarbonate solution containing 5% RapiGest SF Surfactant (Waters) and 2 μL 0.1 mM dithiothreitol at 60°C for 10 minutes. Subsequently, 2 μL of 0.3 mM iodacetamide was added, and the punches were incubated at room temperature in the dark for 30 minutes. Finally, following the addition of 0.5 μg/μL trypsin (Sigma Aldrich), proteolysis was performed at 37°C overnight. The reaction was stopped by adding trifluoroacetic acid to a final concentration of 5%. The samples were centrifuged at room temperature for 15 minutes at 16,000*g*, and the supernatant was recovered and processed using StageTips (Thermo Fisher Scientific) according to the manufacturer's protocol. The resulting peptide solution was lyophilized and stored at −20°C until analysis.

### Targeted Metabolomics

The targeted identification and quantification of nominal 188 metabolites was achieved by executing the mass spectrometric acquisition methods, as outlined by the AbsoluteIDQ kit with some modifications. Mass spectrometric analyses were performed on a 6500 QTRAP (ABSciex, Darmstadt, Germany) coupled with an Eksigent 200 microLC chromatography system (ABSciex). To detect amino acids and biogenic amines, 50 μL of the metabolite extract was diluted in 50 μL of methanol following the addition of 300 μL of H_2_O. Chromatography was performed using a gradient of two running solvents (A: water, 0.2% formic acid; B: acetonitrile, 0.2% formic acid). Two microliters of the diluted metabolite extracts were injected on a BEH C18 column (1.0 × 50 mm/1.7 μm; Waters) and resolved using a linear gradient from 2% solvent B to 40% B in 3.5 minutes, followed by an increase to 80% B in 1.5 minutes, at 30 μL/min, with a final step up to 98% solvent B in 0.1 minutes.

To determine the content of glycerophospholipids, hexoses, and acylcarnitines, 50 μL of the metabolite extract was diluted with 450 μL methanol. Five microliters of this dilution were then analyzed in the mass spectrometer by direct infusion using the acquisition parameters provided in the manufacturer's manual. Two injections were used to acquire data in positive and negative modes, separately.

### Targeted and Shotgun Proteomics

Liquid chromatography tandem mass spectrometry (HPLC-MS/MS) analysis was performed on a NanoRSLC3000 HPLC system (Dionex) coupled to a QExactive plus mass spectrometer (Thermo Fisher Scientific) by a nano spray ion source. Tryptic peptide mixtures derived from the proteomic sample preparation were automatically injected and loaded at a flow rate of 30 μL/min in 98% solvent C (0.1% trifluoroacetic acid in HPLC-grade water) and 2% solvent E (80% actetonitrile and 0.08% formic acid in HPLC-grade water) onto a nano trap column (300 μm inner diameter [i.d.] × 5 mm, packed with Acclaim PepMap100 C18, 5 μm, 100 Å; Dionex). After 3 minutes, peptides were eluted and separated on the analytical column (75 μm i.d. × 25 cm, Acclaim PepMap RSLC C18, 2 μm, 100 Å; Dionex) by a linear gradient from 2% to 30% of solvent E in solvent D (2% acetonitrile and 0.1% formic acid in HPLC-grade water) at a flow rate of 300 nL/min over 82 minutes. The remaining peptides were eluted by a short gradient from 30% to 95% solvent E in 5 minutes. For shotgun proteomics, which was performed on a few selected samples primarily to monitor the influence of the extraction process on the protein identification, the eluted peptides were analyzed in the mass spectrometer using the following acquisition protocol. On the basis of prescans taken at a resolution of 70,000, and covering a mass to charge ratio (m/z) range of 150 to 2000 Da, the 10 most intense at least doubly charged peptide ions were selected for fragment analysis in the quadrupole. The collision energy for higher energy collisional dissociation (HCD) was set to a value of 26, and the resulting fragments were detected with a resolution of 17,500 in the orbitrap. The lock mass option was activated and set to a background signal with a mass of 445.12002.^28^ Every ion selected for fragmentation was excluded for 20 seconds by dynamic exclusion.

Targeted single-ion monitoring (t-SIM) and parallel reaction monitoring (PRM) of the selected proteotypic peptides of 15 proteins (alpha-enolase, extracellular glycoprotein lacritin precursor, hemopexin, lactotransferrin, lipocalin-1, lysozyme C, mammaglobulin, mucin-5AC, myosin 14, prolactin-inducible protein, proline-rich protein 1 precursor, proline-rich protein 4 precursor, retinal dehydrogenase-1, serotransferrin, and 14-3-3 protein zeta/delta) were performed for each sample using the same chromatography conditions as for shotgun proteomics. Therefore, the tryptic peptide mixtures were also injected and loaded on a NanoRSLC3000 HPLC system and analyzed by using the QExactive plus mass spectrometer. The quadrupole mass filter enables the precursor selection of predefined precursor ions, which can be analyzed in the orbitrap. The settings of the mass spectrometer have been optimized using a mixture of synthetic peptide standards that represented proteotypic peptides for the 15 selected tear proteins. For the t-SIM, a scan at a resolution of 70,000 with an injection time of 150 ms, an automatic gain control (AGC) target of 5^e4^ and an isolation window of 4 mass units were defined. Following the targeted filtering, the precursor ions were fragmented in the HCD cell at a collision energy of 26. All resulting fragment ions were analyzed in parallel in the orbitrap at a resolution of 17,500, applying an injection time of 200 ms, an AGC target of 2^e5^, and an isolation window of 2 mass units.

### Data Processing

Mass spectrometric data generated by the metabolomics kit were processed by Analyst software version 1.6 (ABSciex, Darmstadt, Germany) and MetIQ software version 5.5.4-DB100-Boron-2623 (Biocrates Life Sciences, Innsbruck, Austria) according to the kit manual. Tear samples were treated as “serum/plasma” as the kit had not been validated for tear fluid by the manufacturer. As a consequence, the concentration values were incorrect with regard to absolute quantification because the exact volume of tear fluid in a punch was not provided. However, due to normalization of the data to the sum values of each analyte class, this discrepancy was irrelevant for the statistical analysis.

To analyze targeted proteomics data, mass spectra were processed using the open source tool Skyline.^29^ Peptide settings were set as follows: trypsin/P [KR I -] was chosen as the enzyme and one missed cleavage was allowed. Peptides with a sequence length between 6 and 30 amino acids were recognized. For Transition Settings, the allowed charges of precursor ions were set to 2, 3 and the resulting p-, b- and y-ions were chosen for the analysis. The sequences of the predefined precursors (peptide sequences as described in the Chemicals and Reagents section) were imported, and the method was tested using a mixture of the synthetic peptide standards. After processing the raw data, an automated evaluation integration was performed. For further data analysis and statistics, sum values were calculated of the peak areas of the three isotopic precursor masses for all peptides.

For shotgun proteomic analysis, MS raw data were processed using the MaxQuant software (version 1.5.3.3).^30^ Trypsin/P was set as the cleaving enzyme. Cysteine carbamidomethylation was selected as the fixed modification, and both methionine oxidation and protein acetylation were defined as variable modifications. Two missed cleavages per peptide were allowed. The peptide and protein false discovery rates were set to 1%. The initial mass tolerance for precursor ions was set to 7 ppm and the first search option was enabled with 10-ppm precursor mass tolerance. The fragment ion mass tolerance was set to 0.5 Da. The Swiss-Prot_2014 database (selected for *homo sapiens*, 20203 entries) was used for peptide and protein identification. Contaminants like keratins were automatically detected by enabling the MaxQuant contaminant database search. A minimum number of two unique peptides with a minimum length of seven amino acids needed to be detected to perform protein quantification. Only unique peptides were selected for quantification. For label-free quantification (LFQ) the minimum LFQ count was set to two and the requantify option was chosen. The option match between runs was enabled with a time window of 0.7 minutes; fast LFQ was disabled.

### Data Deposition

The shotgun proteomics data have been deposited to the ProteomeXchange Consortium^31^ via the PRIDE partner repository^32^ with the data set identifier PXD008536.

### Statistical Analysis

The statistical analysis included several normalization strategies using the sum of signals of a specific group of measurements (e.g., of metabolite classes) or the accumulated signal intensities of an LC-MS/MS run (total ion count, TIC). Subsequently, three main types of analyses were performed as follows: (1) assessment of intraindividual variability by the calculation of coefficients of variation (CVs) for measurements within the same patient (*n* = number of measurements within a patient). Thus, for each patient, a CV was obtained and the multitude of CVs could be analyzed statistically (*n* = number of subjects). This was done in a subject-related (measurements from left and right eye pooled), or eye-related manner (measurements from both eyes analyzed separately). (2) Assessment of interindividual variability by calculation of CVs for average values of pooled measurements from left and right eye per subject (*n* = number of subjects). (3) Determination of correlation between measurements at left and right eye of the same subject. A nonparametric correlation measure (Spearman correlation coefficient) was chosen, as normal distribution could not be confirmed for each type of measurement, and a consistent approach was preferred for all variables. Formally, the level of significance was set to 0.05 for correlation analyses. However, due to the larger number of variables and small number of subjects, *P* values and significance statements serve only descriptive purposes; no correction for multiple testing was applied. Instead, the entire pattern of correlations among all of the groups of molecules should be noted. In an ex post analysis, for several coefficients of variation the standard errors (SE) were determined according to the formula SE (CV) = Sqrt (CV^2^ × [0.5 + CV^2^]) / (*n* – 1).^33^ Herewith, the confidence interval (CI) can be approximated by CV_mean_ ± 2 × SE. Analysis was performed using commercially available software (Excel Professional Plus 2013, Microsoft Corporation, Redmond, WA; SPSS release 24, IBM, Armonk, NY; MetIDQ version 6.0.0-DB104-Carbon-2743; Biocrates Life Sciences, Innsbruck, Austria).

## Results

### Intraindividual Variation

**Figure 2:**
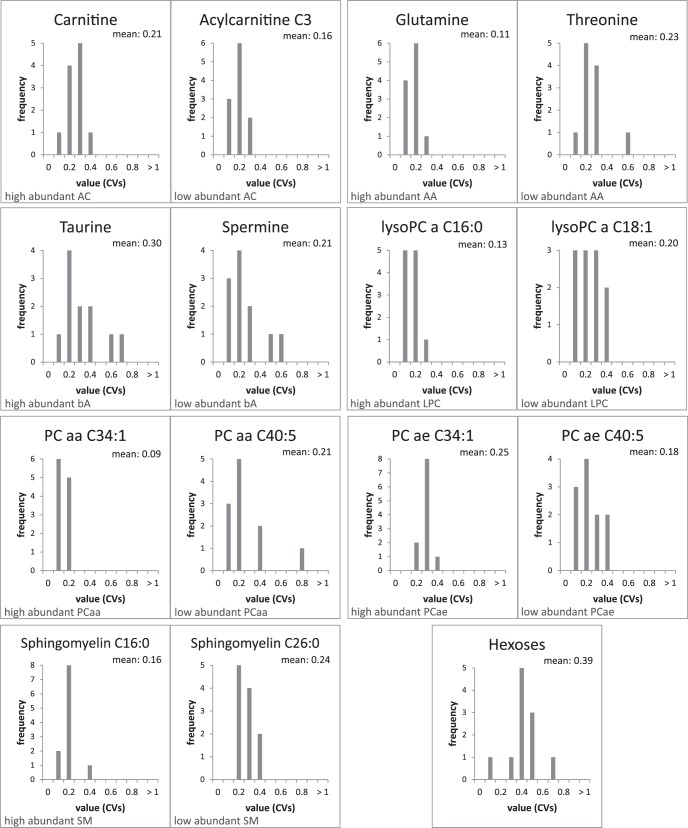
Histograms of intraindividual variability for selected metabolites. CVs have been calculated from six individual samples per subject regardless of the left or right eye. The distribution of frequencies of the CVs is shown for one representative (as indicated by name) of each high abundant and low abundant metabolite of the analyte classes acylcarnitines (AC), amino acids (AA), biogenic amines (bA), lyso-phosphatidylcholines (LPC), diacyl-phosphatidylcholines (PCaa), acyl-alkyl-phosphatidylcholines (PCae), sphingomyelins (SM), and hexoses. In addition, mean values are given for each metabolite.

The ophthalmologic examination of all volunteers revealed that none exhibited any eye-related disease. During the three visits, the study subjects exhibited reproducible tear secretion rates with an average relative standard deviation (CV) of 14% and a mean of 3.7 mm/min, except for those with very high or very low rates. For the outliers, relative standard deviations between 21% and 50% were calculated (Table 1). To investigate the variations of metabolites present in tear fluid, we measured acylcarnitines, amino acids, biogenic amines, lyso- and phosphatidylcholines, sphingomyelins, and hexoses in a targeted fashion using mass spectrometry. Applying the validation criteria of the used method, we were able to detect and quantify 60 metabolites in every sample (Supplementary Table S1), with between 64 and 112 metabolites per sample, although 100 or more metabolites could be quantified in the majority of samples. An overview of the distribution of the detected analytes according to metabolite classes is given in Table 2. On the basis of the quantitative values, we calculated CV values of every determined metabolite for each subject. Histograms of all subject-related CV values were plotted, and average variances for all analytes were calculated to assess analyte-specific differences (Supplementary Table S2). In Figure 2, histograms for the representatives of each analyte class with the highest and lowest abundance are shown. Calculations were performed following normalization to the sum values of the respective analyte class, resulting in relative concentrations for each metabolite. Mean coefficients of variation ranged from 9% to 39%. However, all of the CV's were below 25% for the phospholipids, amino acids, acylcarnitines, and biogenic amines shown; only those for taurine and hexoses were higher (Fig. 2).

**Table 1:**
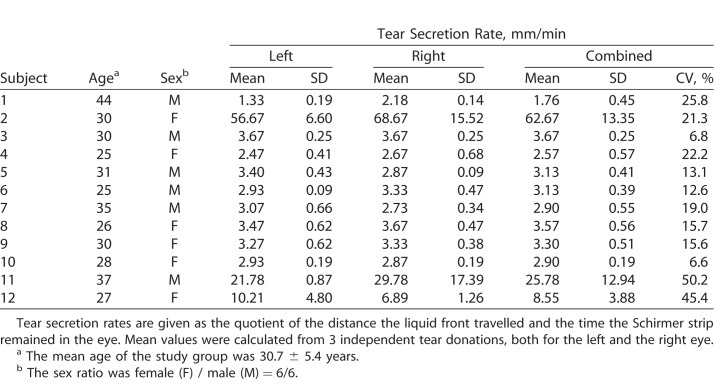
Summary of Epidemiologic Data of the Study Cohort and Determined Tear Secretion Rates

**Table 2:**
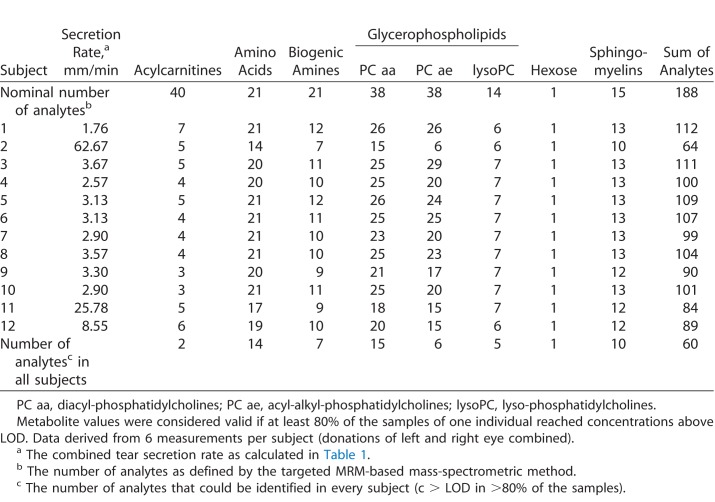
Number of Detectable Metabolites in Tear Fluid Samples by Targeted Metabolome Analysis

Next, we analyzed a selection of 15 proteins, as listed in Table 3, based on physiological and pathological importance. This was done by using the remaining, identical punches of the Schirmer strips, which had already been processed for metabolomics, for limited proteolytic cleavage and quadrupole orbitrap mass spectrometry of selected proteotypic peptides. In contrast to metabolite measurements, variations of intraindividual protein concentrations were generally higher (Table 3). However, when data were normalized to the sum of all targeted signals, the calculated mean variations were improved, particularly for the three most abundant proteins lactotransferrin, lysozyme C, and lipocalin 1, which exhibited mean CV values in a reasonable biological range (29%, 21%, and 15%, respectively). To investigate the general analytic accessibility of proteins in the processed punches we performed classical untargeted proteome analysis. For that purpose, only a subset of the available samples was used, as untargeted proteomics was not the main objective of this work. However, by analyzing at least one sample per subject, we were able to identify up to 685 individual proteins with an average number of 533 identifications across all measured samples (see Table 4 for an overview, and the complete data set in the PRIDE repository). In comparison to the proteomic analysis of nonderivatized samples and punches without pre-extraction, the identification results for the pre-extracted punches were slightly better, as roughly 10% more proteins per sample were identified (Supplementary Table S3).

**Table 3:**
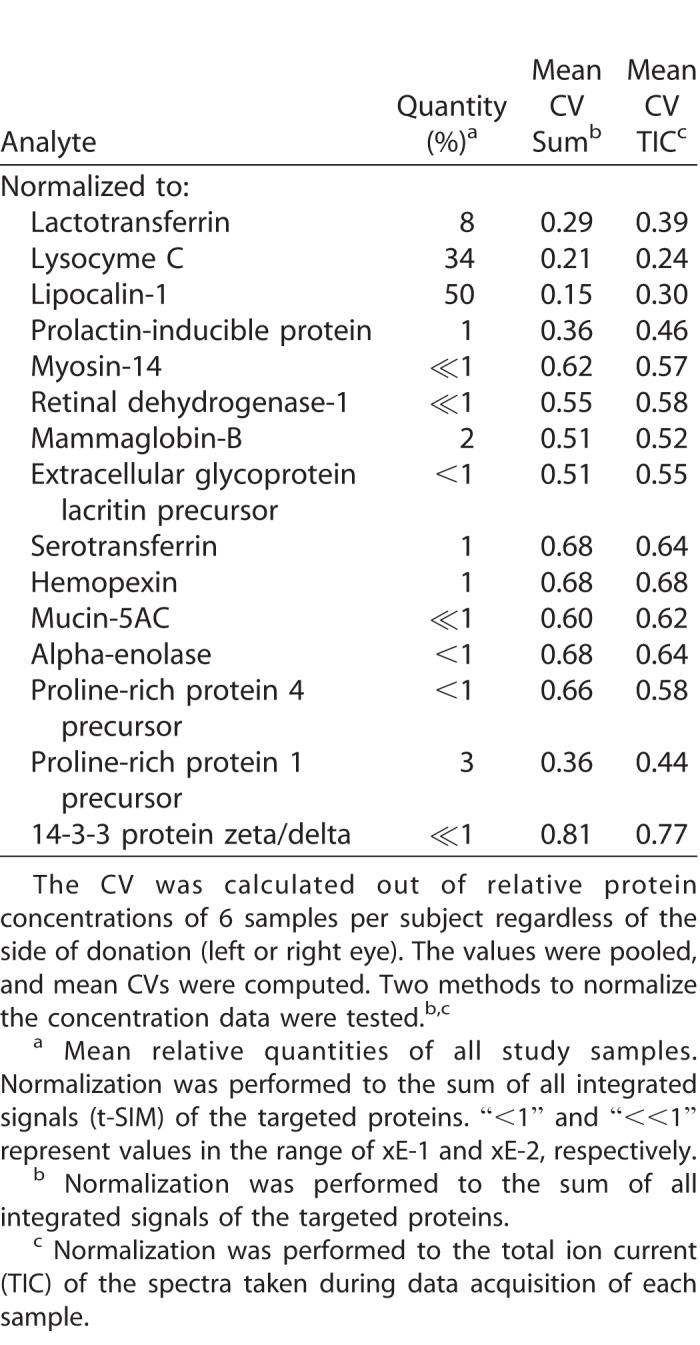
Mean Intraindividual Coefficients of Variation of Relative Protein Concentrations of 12 Individual Subjects

**Table 4:**
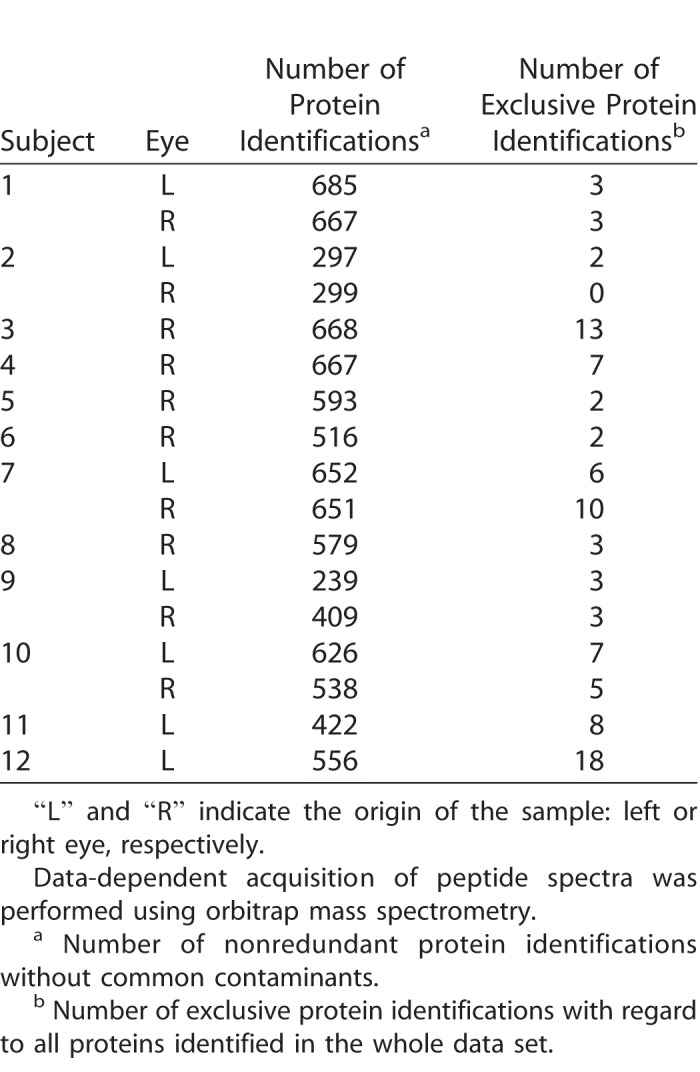
Shotgun Proteomic Analysis of at Least One Representative Tear Sample (Pre-extracted Schirmer Punches) Per Subject

### Evaluation of Tear Fluid Analysis of Left and Right Eye

**Figure 3:**
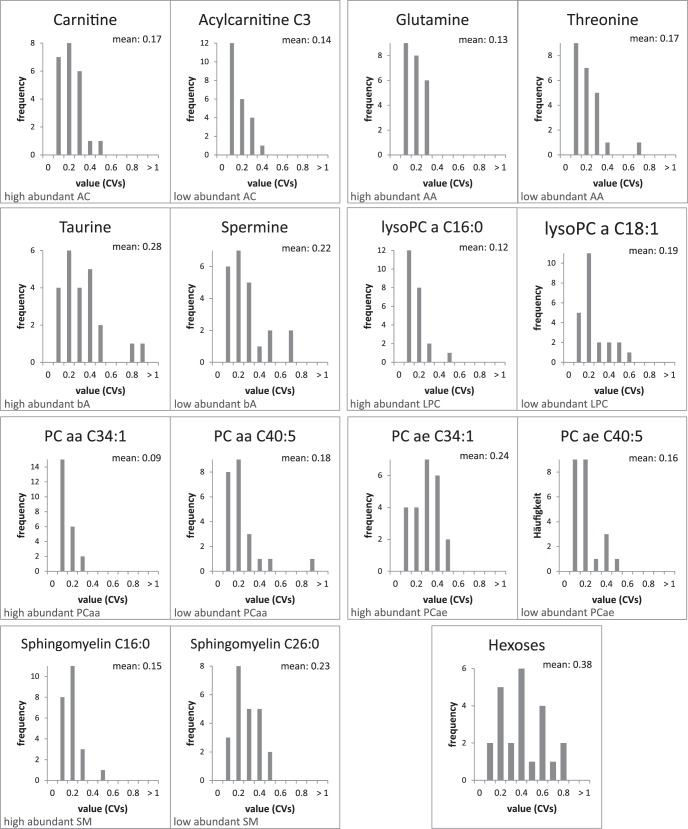
Histograms of intraindividual variability considering individual eyes for selected metabolites. Relative standard deviations (CVs) have been calculated from three individual samples of each left and right eye per subject. The distribution of frequencies of the CVs is shown for one representative (as indicated by name) of each high abundant and low abundant metabolite of the analyte classes AC, AA, bA, LPC, PCaa, PCae, SM, and hexoses. In addition, mean values are given for each metabolite.

To evaluate potential differences between left and right eye our study was designed so that tear fluid was always taken from both (healthy) eyes during one collection date. There was no obvious influence of eye side on the total number of consistently detected metabolites and proteins within eyes. Moreover, the relative distribution of analytes per analyte class did not vary markedly between the two eyes of each volunteer, apart from the general differences caused by different tear secretion rates (Supplementary Fig. S1). Along the lines of the assessment of intraindividual variability, we calculated relative standard deviations for each metabolite of both eyes separately (Supplementary Table S4). An overview of the distribution of CVs considering the side with regard to the highest and lowest abundant metabolite of each analyte class is given in Figure 3. Both for the lowest and highest abundant representative of each analyte class, most of the values were below 25%, and even clearly below 20%, except for taurine (28%) and the pool of hexoses (38%)—as for the intraindividual variation. Furthermore, we performed a correlation analysis of the analyte concentrations between the individual results of left and right eye. Table 5 summarizes the results of the correlation analysis with regard to the highest and lowest abundant representative of each analyte class. Additionally, the coefficients were calculated with both nonnormalized and normalized concentration data. It is noteworthy that the analyses of proteins also reflected such a high level of correlation between tear fluids of the left and right eyes—for the targeted quantification as well as for the sheer number of protein identifications derived from the shotgun approach performed on a few random samples.

**Table 5:**
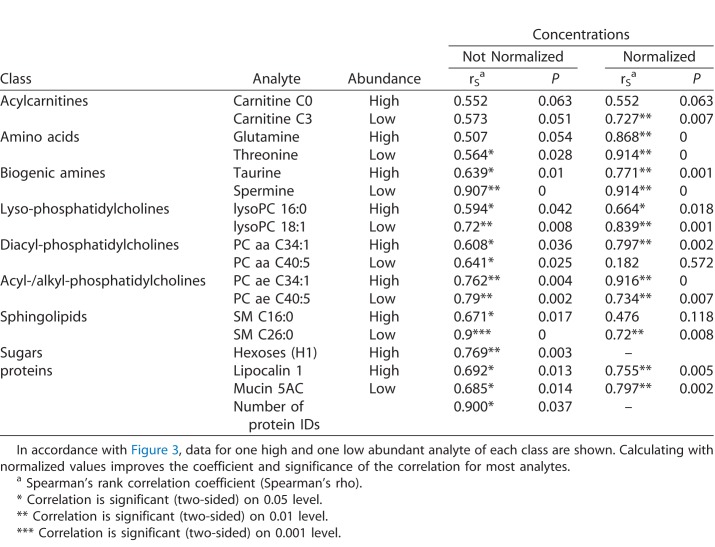
Nonparametric Correlation Analysis of Individual Analyte Concentrations (With and Without Normalization) in Tear Fluid Taken From the Left and the Right Eye of the Study Subjects

### Interindividual Variation

**Figure 4:**
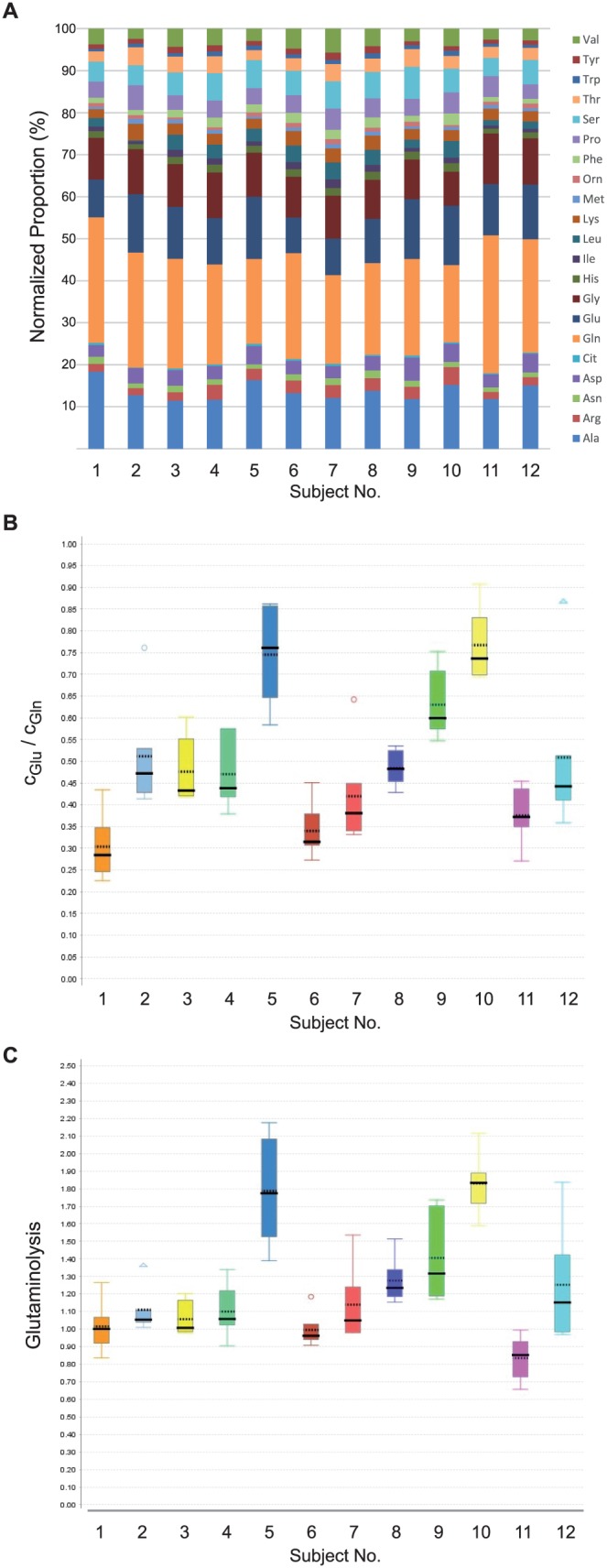
Interindividual variations of selected metabolites and metabolite ratios regarding the study cohort. (A) Average normalized concentrations of amino acids have been calculated for each patient, and are displayed in a proportional bar chart to allow comparison of the individual subjects at a glance (colors represent single amino acids, as indicated by the legend). (B) Glu/Gln was calculated of absolute amino acid concentrations. The resulting values are shown as box blots for each subject numbered 1 to 12. ANOVA was computed and revealed significant statistical differences within the cohort (data not shown). (C) A surrogate parameter for glutaminolysis, namely the ratio of the sum of amino acid concentrations of aspartate, alanine, and glutamate to the concentration of glutamine was calculated. The resulting values are shown as box blots for each subject (numbers 1–12). ANOVA was computed and revealed significant statistical differences within the cohort (q < 0.001, data not shown). Values of subjects 5 and 10 are significantly different from the rest as revealed by Student's t-test.

During sample taking, we used our specially designed Schirmer strips, a procedure that is well tolerated by the donors in most cases. However, subject 2 showed very strong reflex tear secretion, and two other subjects (11 and 12) had elevated secretion rates compared with the average rates of the remaining subjects, as summarized in Table 1. In donors with secretion rates above 20 mm/min, the concentration values dropped below the limit of detection (LOD) for up to 30% of the major metabolites (e.g., amino acids). Moreover, for the three volunteers mentioned above, sample taking had to be stopped in accordance with the study protocol even before reaching the 5-minutes sampling time. Given such extreme differences in tear secretion, we sought to investigate the influence of individual secretion rates on the number of detectable analytes first. In the case of reflex tears, the ratio of individual constituents should be considered to be at least partially disturbed in favor of a volume-increasing aqueous matrix. In fact, the number of metabolites that could be detected at concentrations above the LOD dropped for the samples of subject 2 by almost half in comparison to the average. Protein concentrations were stable across the cohort in the case of abundant proteins, whereas the concentrations of low abundant proteins were preferentially affected by very low tear secretion rates (Supplementary Fig. S2). Nevertheless, we calculated the overall variances for each analyte on the basis of the normalized data and mean concentrations for the entire cohort as already shown in Supplementary Table S1. A graphic overview of the interindividual distribution of amino acids of all study subjects is given in Figure 4A, and visualization of the protein data is provided in Supplementary Figure S2.

To approximate the CIs of our findings, we have calculated the standard errors for the relevant coefficients of variation and number of samples (Supplementary Table S5). Next, the question of whether there were particular outliers in the study cohort was asked. This involved looking at metabolite ratios in physiological homeostasis, for example, the glutamate/glutamine (Glu/Gln) ratio, which should not be strongly affected by the tear secretion rate, as they are supposed to be stable in healthy individuals. By plotting the Glu/Gln ratio, it was demonstrated that there were only a few subjects who exhibited substantially different values with regard to the average (Fig. 4B). Taking additional analyte concentrations into account, for instance, for the description of glutaminolysis in a biological system ([c_Asp_ + c_Ala_ + c_Glu_]/c_Gln_), it was possible to reveal the individual nature of metabolite profiles, at least for some particular subjects. In this case, subjects 5 and 10 exhibited significantly different mean ratios than the rest of the cohort, as shown in Figure 4C.

## Discussion

In our workflow we used mass-spectrometric detection based on multiple reaction monitoring (MRM) targeting a range of more than 150 housekeeping metabolites (acylcarnitines, amino acids, taurine, sphingomyelins, glycerophospholipids, etc.), and t-SIM determining 15 tear proteins (lipocalin 1, lactotransferrin, prolactin-inducible protein, alpha-enolase, etc.), which were reported to be relevant for ocular diseases.^9,11,22,27^ It is important to mention that both analyses were done from the identical punches of Schirmer strips, with which the tear samples had been collected from the subjects (Fig. 1).

We were able to determine an average of approximately 104 metabolites (60 could be determined in every subject), and the 15 selected proteins in a quantitative or semiquantitative way, whereas the absolute number primarily depended on the extremes of individual tear secretion rates. In this context, the values for up to 30% of key metabolites (e.g., amino acids) dropped below the limit of detection for donors with secretion rates above 20 mm/min, remarkably not affecting all analyte classes to the same extent. Whereas the number of phosphatidylcholines dropped dramatically, and the groups of amino acids and biogenic amines were the second most affected, the total numbers of detectable lyso-phosphatidylcholines, sphingomyelins, and acylcarnitines were rather unaffected. Therefore, we generally normalized the concentration values according to sum values of each analyte class improving variability and comparability substantially.

In addition, we detected low abundance proteins that did not behave according to a simple dilution effect as well (e.g., mammaglobulin and mucin 5A). At least concerning mucin 5A, it is not surprising that it could be detected in every sample because it might be transferred to the Schirmer strip physically rather than by active tear secretion, as the almost insoluble mucin 5A is sheared from the epithelial glycocalyx.^1^ This exemplifies the fact that detailed physiological analyses on an analyte-to-analyte basis would be necessary to investigate whether the differential behavior of certain analytes happens due to passive or active secretion processes in the tear and Meibomian glands. Concerning future study design, these findings strongly suggest to consider the stratification of subjects according to tear secretion rate before enrolling them.

Intraindividual variation with regard to the time of sample donation, and thus to the nutritional status, was in the range of 10% to 25% (at a reasonable confidence interval of ±4%–10% for 12 subjects) except for the major energy donor sugar, and the homeostatic compound taurine. It is not surprising that both taurine, in its role as a major osmotic regulator, and hexose, which consists of more than 98% glucose in human biofluids, were highly variable in tears as they are directly linked to energy homeostasis. Nevertheless, the results favor open study regimes and the applicability in health care centers, although further studies are necessary to investigate all potential environmental influences.

Although most of the 15 selected proteins could be detected in the samples from almost every subject, the proteomic results suggest that the low abundance proteins are more difficult to quantify in the presence of abundant ones, as the overall concentration range is broader than that of the metabolites. Moreover, we assume that the elevated variations in protein concentrations in contrast to those of metabolite concentrations were influenced by the unusual sample preparation method (i.e., after an additional derivatization step) and directly in the filter punches in contrast to other proteomic studies so far.^24^ Most likely the enzymatic access has been impaired. For future studies, we suggest to add optimized isotope-labelled peptides or proteins as internal standards before executing the proteomics part of the method. In contrast, identifying 533 unique proteins in the Schirmer punches by shotgun proteomics was quite remarkable in comparison to earlier reports of studies that have been primarily designed for proteomics.^9,34^ Comparing our proteomics results with and without preceding metabolomics sample preparation, the chemical modification introduced by the metabolomics step did not dramatically influence the identification rate. On the contrary, the pre-extraction seemed to have a beneficial effect, probably due to the removal of lipids, which can influence proteomic analysis.^35^

For the majority of analytes (>80%), the correlation between both (healthy) eyes was significant with correlation coefficients more than 0.5, in some cases even more than 0.8, while the intraindividual variations were exhibiting similar values in comparison to the situation without considering the particular eye of the subjects. High physiological correlation between both eyes has been shown for some other ocular parameters before as for tear osmolarity in a study with dry-eye patients, and similar congruency was previously reported for lens density and thickness of the retinal nerve fiber layers.^36–38^

With regard to interindividual variation, two major factors need to be taken into account concerning the design and execution of future clinical studies. First, as mentioned before, the individual tear secretion rate has an impact on the concentration data and therefore might require patient stratification. Second, the study data seem to support the hypothesis that individual tear metabolite patterns exist, as some findings regarding specifically influenced analytes or even analyte ratios correlated to neither the demographic parameters nor the individual tear secretion rates of subjects (Table 1). It is most likely that individual phenotypes could also be detected in tear fluid, as such findings have been reported for other samples emitted by humans (e.g., exhaled breath and saliva).^39–41^ We hypothesize that influencing factors like diet or menstrual cycle could also be identified for the individual healthy tear metabolome if the number of measured epidemiologic parameters and study size were considerably increased.

In conclusion, a detailed investigation of the applicability of targeted metabolomics and proteomics of tear fluid led to a workflow that is potentially applicable in clinical practice. Both sample taking with Schirmer strips and handling for medical staff is rather easy as it includes simple air-drying and storage in a freezer. The sample preparation for mass-spectrometric analysis is standardized by using defined punches of the strips and a consecutive analyte extraction process that employs a commercially available kit. Although our study cohort was too small to gain valid insight into individual tear homeostasis and bioprofiles, the method could be well integrated in protocols of larger studies, either to describe molecular changes during pathogenesis, identify biomarker patterns, or investigate the effects of therapeutic intervention.

## Supplementary Material

Supplement 1

Supplement 2

Supplement 3

Supplement 4

Supplement 5

Supplement 6

Supplement 7
