# Supplementary material for: Combined Targeted Analysis of Metabolites and Proteins in Tear Fluid With Regard to Clinical Applications
Source: Transl Vis Sci Technol. 2018 Dec 6;7(6):22. doi: 10.1167/tvst.7.6.22 (PMC6284467; doi:10.1167/tvst.7.6.22)
Supplement: Supplement 1 [file tvst-07-06-18_s01.pdf]

**Title:** Combined Targeted Analysis of Metabolites and Proteins in Tear Fluid with Regard to Clinical Applications

**Jounal:** TVST

**Authors:** Sascha Dammeier, Peter Martus, Franziska Klose, Michael Seid, Dario Bosch, Janina D‘Alvise, Focke Ziemssen, Spyridon Dimopoulos and Marius Ueffing

**Corresponding Author:** Sascha Dammeier, Institute for Ophthalmic Research, Core Facility for Medical Bioanalytics, University of Tübingen, Elfriede-Aulhorn-Strasse 7, 72076 Tübingen, Germany, email: sascha.dammeier@uni-tuebingen.de

**SUPPLEMENTARY TABLE S1.** Inter-individual variation of quantified metabolites and proteins in the study cohort. Raw data of all study samples were filtered according to the presence of valid values in at least 80 % of the subjects. The data were normalized to sum values of each analyte class. Means, standard deviations and coefficients of variation were calculated for each analyte except for hexoses, for which absolute values were taken. Green coloring indicates analytes that could be found in every sample allowing a maximum of one outlier per subject, e.g. exhibiting a concentration value below limit of detection.

| Acylcarnitines | mean | SD | CV |
|---|---|---|---|
| C0 | 66.98 | 6.40 | 0.10 |
| C2 | 23.66 | 4.59 | 0.19 |
| C3 | 4.96 | 2.71 | 0.55 |
| C5 | 4.40 | 2.76 | 0.63 |
| Amino Acids | | | |
| Ala | 13.67 | 2.47 | 0.18 |
| Arg | 2.62 | 0.99 | 0.38 |
| Asn | 1.40 | 0.32 | 0.23 |
| Asp | 3.70 | 1.12 | 0.30 |
| Cit | 0.40 | 0.22 | 0.54 |
| Gln | 24.77 | 4.82 | 0.19 |
| Glu | 11.89 | 2.72 | 0.23 |
| Gly | 10.17 | 1.49 | 0.15 |
| His | 1.65 | 0.37 | 0.23 |
| Ile | 1.27 | 0.48 | 0.38 |
| Leu | 2.72 | 1.35 | 0.50 |
| Lys | 2.84 | 0.73 | 0.26 |
| Met | 0.82 | 0.24 | 0.30 |

| | mean | SD | CV |
|---|---|---|---|
| Orn | 0.88 | 0.44 | 0.50 |
| Phe | 1.82 | 0.65 | 0.36 |
| Pro | 4.36 | 1.01 | 0.23 |
| Ser | 5.82 | 1.67 | 0.29 |
| Thr | 3.33 | 0.77 | 0.23 |
| Trp | 0.95 | 0.27 | 0.29 |
| Tyr | 1.25 | 0.32 | 0.26 |
| Val | 3.70 | 1.08 | 0.29 |
| Biogenic Amines | | | |
| ADMA | 0.01 | 0.02 | 1.22 |
| SDMA | 0.01 | 0.01 | 0.80 |
| alpha-AAA | 0.12 | 0.13 | 1.02 |
| Carnosine | 0.02 | 0.03 | 2.12 |
| Creatinine | 3.48 | 4.93 | 1.42 |
| Met-SO | 0.05 | 0.06 | 1.07 |
| Putrescine | 0.23 | 0.12 | 0.53 |
| Sarcosine | 3.38 | 5.18 | 1.53 |
| Spermidine | 0.17 | 0.09 | 0.51 |

| | mean | SD | CV |
|---|---|---|---|
| Spermine | 0.69 | 0.36 | 0.53 |
| t4-OH-Pro | 0.60 | 0.85 | 1.43 |
| Taurine | 92.53 | 2.74 | 0.03 |
| Lyso-PC | | | |
| lysoPC a C16:0 | 55.97 | 7.46 | 0.13 |
| lysoPC a C16:1 | 1.40 | 0.28 | 0.20 |
| lysoPC a C17:0 | 1.51 | 0.26 | 0.17 |
| lysoPC a C18:0 | 18.02 | 3.25 | 0.18 |
| lysoPC a C18:1 | 16.50 | 2.89 | 0.18 |
| lysoPC a C18:2 | 6.07 | 2.97 | 0.49 |
| lysoPC a C20:4 | 0.53 | 0.29 | 0.55 |
| Hexoses | | | |
| Hexoses* (absolute) | 228.45 | 121.57 | 0.53 |
| Hexoses† (normalized to AA) | 50.19 | 19.99 | 0.40 |

| PC aa | mean | SD | CV |
|---|---|---|---|
| PC aa C28:1 | 0.31 | 0.23 | 0.76 |
| PC aa C30:0 | 1.23 | 0.56 | 0.46 |
| PC aa C32:0 | 5.86 | 1.62 | 0.28 |
| PC aa C32:1 | 1.55 | 0.23 | 0.15 |
| PC aa C32:2 | 0.20 | 0.06 | 0.29 |
| PC aa C34:1 | 26.20 | 3.08 | 0.12 |
| PC aa C34:2 | 20.25 | 2.36 | 0.12 |
| PC aa C34:3 | 0.67 | 0.15 | 0.23 |
| PC aa C34:4 | 0.06 | 0.03 | 0.50 |
| PC aa C36:1 | 5.85 | 1.17 | 0.20 |
| PC aa C36:2 | 14.79 | 1.32 | 0.09 |
| PC aa C36:3 | 7.74 | 1.54 | 0.20 |
| PC aa C36:4 | 5.54 | 1.24 | 0.22 |
| PC aa C36:5 | 0.39 | 0.11 | 0.29 |
| PC aa C36:6 | 0.06 | 0.04 | 0.63 |
| PC aa C38:0 | 0.28 | 0.16 | 0.58 |
| PC aa C38:3 | 1.51 | 0.26 | 0.17 |
| PC aa C38:4 | 3.11 | 0.71 | 0.23 |

| | mean | SD | CV |
|---|---|---|---|
| PC aa C38:5 | 1.59 | 0.31 | 0.20 |
| PC aa C38:6 | 1.32 | 0.33 | 0.25 |
| PC aa C40:3 | 0.09 | 0.05 | 0.58 |
| PC aa C40:4 | 0.15 | 0.05 | 0.36 |
| PC aa C40:5 | 0.19 | 0.05 | 0.29 |
| PC aa C40:6 | 0.99 | 0.48 | 0.48 |
| PC aa C42:1 | 0.07 | 0.07 | 0.99 |
| PC ae | | | |
| PC ae C32:1 | 4.07 | 0.87 | 0.21 |
| PC ae C34:0 | 4.80 | 1.09 | 0.23 |
| PC ae C34:1 | 14.69 | 2.19 | 0.15 |
| PC ae C34:2 | 9.58 | 1.78 | 0.19 |
| PC ae C34:3 | 3.64 | 0.91 | 0.25 |
| PC ae C36:1 | 12.62 | 2.50 | 0.20 |
| PC ae C36:2 | 11.20 | 1.67 | 0.15 |
| PC ae C36:3 | 3.89 | 0.91 | 0.23 |
| PC ae C36:4 | 5.43 | 1.12 | 0.21 |
| PC ae C36:5 | 5.34 | 1.54 | 0.29 |

| | mean | SD | CV |
|---|---|---|---|
| PC ae C38:2 | 2.37 | 0.71 | 0.30 |
| PC ae C38:3 | 2.29 | 0.41 | 0.18 |
| PC ae C38:4 | 4.70 | 0.91 | 0.19 |
| PC ae C38:5 | 5.39 | 1.07 | 0.20 |
| PC ae C38:6 | 2.00 | 0.62 | 0.31 |
| PC ae C40:1 | 1.36 | 0.65 | 0.48 |
| PC ae C40:2 | 2.24 | 0.47 | 0.21 |
| PC ae C40:3 | 1.28 | 0.34 | 0.26 |
| PC ae C40:5 | 1.30 | 0.27 | 0.21 |
| PC ae C40:6 | 1.81 | 0.59 | 0.32 |
| SM | | | |
| SM (OH) C14:1 | 2.64 | 0.89 | 0.34 |
| SM (OH) C16:1 | 1.57 | 0.34 | 0.21 |
| SM (OH) C22:1 | 3.21 | 0.57 | 0.18 |
| SM (OH) C22:2 | 1.13 | 0.26 | 0.23 |
| SM (OH) C24:1 | 1.63 | 0.37 | 0.23 |
| SM C16:0 | 58.20 | 2.54 | 0.04 |
| SM C16:1 | 2.04 | 0.45 | 0.22 |

| SM (ctnd.) | mean | SD | CV |
|---|---|---|---|
| SM C18:0 | 6.05 | 0.83 | 0.14 |
| SM C18:1 | 1.08 | 0.33 | 0.31 |
| SM C24:0 | 10.48 | 1.57 | 0.15 |
| SM C24:1 | 10.27 | 1.37 | 0.13 |
| SM C26:0 | 1.14 | 0.39 | 0.35 |
| SM C26:1 | 0.55 | 0.14 | 0.26 |

| Proteins | mean | SD | CV |
|---|---|---|---|
| Lactotransferrin | 7.90 | 2.45 | 0.31 |
| Lysocym C | 33.78 | 9.17 | 0.27 |
| Lipocalin-1 | 49.90 | 9.49 | 0.19 |
| Prolactin-inducible protein | 1.02 | 0.57 | 0.57 |
| Myosin-14 | 0.06 | 0.07 | 1.02 |
| Retinal Dehydrogenase-1 | 0.08 | 0.08 | 1.05 |
| Mammaglobin-B | 2.17 | 1.74 | 0.80 |
| Extracellular glycoprotein lacritin precursor | 0.34 | 0.37 | 1.09 |
| Serotransferrin | 0.53 | 0.66 | 1.23 |
| Hemopexin | 0.60 | 0.66 | 1.10 |
| Mucin-5AC | 0.03 | 0.04 | 1.08 |
| Alpha-Enolase | 0.38 | 0.54 | 1.40 |
| Proline-rich protein 4 precursor | 0.24 | 0.59 | 2.39 |
| Proline-rich protein 1 precursor | 2.89 | 1.94 | 0.67 |
| 14-3-3 protein zeta/delta | 0.08 | 0.11 | 1.39 |

[*] In case of hexoses absolute concentrations (µM/punch) were used for calculations

[†] Theoretical consideration: Relative concentration values for hexoses after normalization to the sum values of amino acid concentrations
