# Supplementary material for: Combined Targeted Analysis of Metabolites and Proteins in Tear Fluid With Regard to Clinical Applications
Source: Transl Vis Sci Technol. 2018 Dec 6;7(6):22. doi: 10.1167/tvst.7.6.22 (PMC6284467; doi:10.1167/tvst.7.6.22)
Supplement: Supplement 2 [file tvst-07-06-18_s02.pdf]

Title: Combined Targeted Analysis of Metabolites and Proteins in Tear Fluid with Regard to Clinical Applications
Jounal: TVST
Authors: Sascha Dammeier, Peter Martus, Franziska Klose, Michael Seid, Dario Bosch, Janina D'Alvise, Focke Ziemssen, Spyridon Dimopoulos and Marius Ueffing
Corresponding Author: Sascha Dammeier, Institute for Ophthalmic Research, Core Facility for Medical Bioanalytics, University of Tübingen, Elfriede-Aulhorn-Strasse 7, 72076 Tübingen, Germany, email: sascha.dammeier@uni-tuebingen.de

Supplementary Table S2. Intra-individual variations of all analytes according to subjects. Coefficients of variation were calculated from normalized concentration values of each subject (6 tear fluid donations)
Red numbers indicate imputed values.

Subject	Amino Acids																				
	**Ala**	**Arg**	**Asn**	**Asp**	**Cit**	**Gln**	**Glu**	**Gly**	**His**	**Ile**	**Leu**	**Lys**	**Met**	**Orn**	**Phe**	**Pro**	**Ser**	**Thr**	**Trp**	**Tyr**	**Val**
1	0.12	0.25	0.14	0.34	0.43	0.12	0.18	0.18	0.34	0.36	0.27	0.10	0.33	0.20	0.22	0.11	0.07	0.20	0.15	0.29	0.36
2	0.12	0.18	0.36	0.16	0.96	0.10	0.14	0.14	0.39	1.31	0.32	0.40	0.30	0.14	0.12	0.13	0.11	0.33	0.25	0.17	0.38
3	0.09	0.22	0.18	0.28	0.34	0.08	0.12	0.18	0.27	0.26	0.25	0.19	0.37	0.17	0.14	0.19	0.15	0.19	0.20	0.24	0.51
4	0.09	0.22	0.22	0.14	0.35	0.10	0.14	0.09	0.27	0.27	0.14	0.11	0.21	0.18	0.07	0.10	0.12	0.10	0.14	0.20	0.55
5	0.17	0.50	0.19	0.32	0.52	0.23	0.18	0.28	0.21	0.59	0.31	0.18	0.25	0.54	0.39	0.30	0.21	0.58	0.14	0.20	0.34
6	0.06	0.20	0.04	0.17	0.08	0.07	0.18	0.10	0.13	0.22	0.17	0.13	0.24	0.22	0.09	0.20	0.07	0.14	0.08	0.07	0.22
7	0.07	0.10	0.11	0.16	0.27	0.14	0.15	0.09	0.23	0.22	0.09	0.13	0.29	0.08	0.08	0.08	0.15	0.13	0.19	0.17	0.70
8	0.12	0.28	0.26	0.18	0.40	0.10	0.07	0.12	0.20	0.26	0.27	0.08	0.20	0.23	0.15	0.13	0.11	0.28	0.14	0.23	0.22
9	0.17	0.61	0.07	0.32	1.12	0.12	0.10	0.44	0.16	0.32	0.14	0.21	0.62	0.29	0.22	0.66	0.20	0.19	0.21	0.15	0.20
10	0.10	0.27	0.09	0.21	0.24	0.04	0.13	0.11	0.13	0.21	0.14	0.13	0.17	0.25	0.22	0.25	0.10	0.21	0.11	0.17	0.23
11	0.10	0.22	0.15	0.15	0.35	0.07	0.14	0.20	0.36	0.48	0.38	0.35	0.41	0.19	0.44	0.10	0.12	0.29	0.24	0.29	0.71
12	0.16	0.26	0.09	0.33	0.52	0.14	0.19	0.46	0.22	0.25	0.19	0.21	1.19	0.16	0.19	0.56	0.24	0.10	0.10	0.14	0.50
mean	0.11	0.28	0.16	0.23	0.47	0.11	0.14	0.20	0.24	0.40	0.22	0.18	0.38	0.22	0.20	0.23	0.14	0.23	0.16	0.19	0.41
SD	0.03	0.13	0.09	0.08	0.28	0.04	0.03	0.12	0.08	0.30	0.09	0.09	0.27	0.11	0.11	0.18	0.05	0.13	0.05	0.06	0.18
CV	0.30	0.48	0.55	0.33	0.61	0.40	0.24	0.63	0.34	0.75	0.39	0.51	0.71	0.50	0.58	0.78	0.39	0.56	0.33	0.32	0.43

Subject	Acylcarnitines																				
	**C0**	**C10**	**C101**	**C102**	**C12**	**C12DC**	**C121**	**C14**	**C141**	**C141OH**	**C142**	**C142OH**	**C16**	**C16OH**	**C161**	**C161OH**	**C162**	**C162OH**	**C18**	**C181**	**C181OH**
1	0.29	0.27	0.40	0.31	0.34	0.30	0.27	0.39	0.42	0.60	0.37	0.13	0.29	0.31	0.37	0.42	0.31	0.28	0.39	0.36	0.17
2	0.28	0.36	0.26	0.35	0.35	0.32	0.25	0.26	0.36	0.38	0.33	0.25	0.24	0.29	0.40	0.43	0.23	0.43	0.28	0.28	0.43
3	0.08	0.10	0.10	0.15	0.14	0.14	0.17	0.14	0.18	0.16	0.13	0.10	0.13	0.16	0.19	0.17	0.20	0.14	0.08	0.22	0.25
4	0.15	0.19	0.16	0.18	0.18	0.12	0.13	0.18	0.14	0.25	0.15	0.12	0.15	0.15	0.21	0.20	0.22	0.27	0.08	0.12	0.16
5	0.20	0.40	0.24	0.28	0.20	0.30	0.30	0.31	0.29	0.23	0.22	0.24	0.26	0.21	0.32	0.22	0.25	0.24	0.69	0.71	0.23
6	0.12	0.09	0.10	0.10	0.08	0.11	0.13	0.14	0.05	0.18	0.17	0.15	0.15	0.15	0.31	0.14	0.27	0.09	0.19	0.18	0.15
7	0.21	0.23	0.21	0.21	0.21	0.27	0.17	0.32	0.31	0.33	0.20	0.20	0.35	0.15	0.20	0.20	0.18	0.24	0.68	0.69	0.09
8	0.15	0.24	0.19	0.18	0.20	0.22	0.18	0.14	0.14	0.23	0.16	0.21	0.22	0.15	0.19	0.28	0.25	0.16	0.23	0.19	0.11
9	0.24	0.38	0.18	0.26	0.21	0.33	0.24	0.25	0.28	0.18	0.19	0.21	0.23	0.27	0.16	0.16	0.28	0.21	0.18	0.14	0.19
10	0.15	0.18	0.11	0.12	0.17	0.20	0.14	0.18	0.10	0.16	0.20	0.13	0.09	0.08	0.21	0.22	0.19	0.13	0.23	0.15	0.20
11	0.37	0.34	0.37	0.33	0.32	0.35	0.43	0.34	0.31	0.36	0.36	0.35	0.39	0.40	0.29	0.32	0.39	0.31	0.49	0.44	0.34
12	0.28	0.31	0.22	0.35	0.23	0.29	0.30	0.33	0.15	0.21	0.46	0.23	0.26	0.34	0.37	0.33	0.25	0.45	0.22	0.24	0.27
mean	0.21	0.26	0.21	0.24	0.22	0.24	0.23	0.25	0.23	0.27	0.24	0.19	0.23	0.22	0.27	0.26	0.25	0.25	0.31	0.31	0.21
SD	0.08	0.10	0.09	0.09	0.08	0.08	0.09	0.09	0.11	0.12	0.10	0.07	0.08	0.09	0.08	0.10	0.06	0.11	0.20	0.20	0.09
CV	0.38	0.39	0.44	0.36	0.35	0.33	0.38	0.35	0.49	0.45	0.42	0.36	0.37	0.42	0.30	0.37	0.22	0.44	0.64	0.62	0.43

Subject	Acylcarnitines																		
	**C182**	**C2**	**C3**	**C3DCC4OH**	**C3OH**	**C31**	**C4**	**C41**	**C6C41DC**	**C5**	**C5MDC**	**C5OHC3DCM**	**C51**	**C51DC**	**C5DCC6OH**	**C61**	**C7DC**	**C8**	**C9**
1	0.21	0.20	0.21	0.31	0.45	0.07	0.40	0.23	0.23	0.68	0.65	0.29	0.44	0.45	0.33	0.30	0.34	0.37	0.58
2	0.20	0.22	0.29	0.31	0.22	0.15	0.32	0.22	0.23	0.37	0.44	0.27	0.32	0.27	0.25	0.32	0.31	0.33	0.29
3	0.04	0.09	0.09	0.10	0.08	0.09	0.12	0.12	0.16	0.47	0.66	0.12	0.21	0.19	0.14	0.22	0.15	0.13	0.31
4	0.11	0.08	0.13	0.20	0.20	0.06	0.16	0.18	0.17	0.40	0.48	0.10	0.23	0.17	0.18	0.23	0.12	0.23	0.32
5	0.08	0.15	0.15	0.10	0.21	0.20	0.18	0.21	0.18	0.34	0.42	0.12	0.27	0.17	0.14	0.16	0.24	0.39	0.33
6	0.18	0.17	0.17	0.19	0.20	0.13	0.08	0.13	0.07	0.38	0.62	0.13	0.16	0.14	0.13	0.27	0.16	0.22	0.29
7	0.19	0.27	0.24	0.19	0.23	0.10	0.42	0.30	0.68	0.61	0.77	0.27	0.33	0.31	0.21	0.28	0.20	0.27	0.29
8	0.13	0.20	0.07	0.13	0.18	0.07	0.14	0.11	0.15	0.51	0.60	0.13	0.21	0.15	0.13	0.18	0.19	0.19	0.34
9	0.20	0.31	0.17	0.21	0.22	0.11	0.16	0.15	0.12	0.34	0.43	0.19	0.20	0.21	0.23	0.26	0.29	0.17	0.31
10	0.09	0.34	0.08	0.15	0.12	0.12	0.22	0.11	0.36	0.31	0.36	0.12	0.18	0.16	0.19	0.24	0.14	0.17	0.35
11	0.24	0.46	0.16	0.27	0.35	0.11	0.33	0.23	0.47	0.51	0.67	0.28	0.34	0.30	0.38	0.36	0.32	0.35	0.26
12	0.12	0.25	0.18	0.30	0.29	0.08	0.22	0.25	0.14	0.14	0.41	0.21	0.14	0.22	0.30	0.42	0.34	0.23	0.28
mean	0.15	0.23	0.16	0.20	0.23	0.11	0.23	0.19	0.25	0.42	0.54	0.19	0.25	0.23	0.22	0.27	0.23	0.25	0.33
SD	0.06	0.10	0.06	0.07	0.09	0.04	0.11	0.06	0.17	0.14	0.13	0.07	0.08	0.09	0.08	0.07	0.08	0.08	0.08
CV	0.40	0.45	0.38	0.36	0.41	0.35	0.47	0.32	0.68	0.33	0.23	0.38	0.34	0.38	0.36	0.26	0.34	0.33	0.24

Subject	Biogenic Amines																	Sugars
	AcOrn	ADMA	c4OHPro	Carnosine	Creatinine	Dopamine	Histamine	Kynurenine	MetSO	PEA	Putrescine	Serotonin	Spermidine	Spermine	t4OHPro	Taurine	SDMA	Hexose
1	0.50	0.52	0.36	0.35	1.10	1.28	0.36	0.19	2.45	0.28	0.20	0.21	0.23	0.03	0.17	0.15	0.09	0.48
2	2.45	0.39	1.00	0.37	1.70	2.45	0.45	0.81	2.45	0.31	0.28	0.36	0.48	0.21	0.12	0.60	0.19	0.43
3	0.35	0.22	2.06	0.20	1.00	2.45	0.09	0.24	1.00	0.37	0.42	0.23	0.12	0.07	0.14	0.24	0.05	0.37
4	0.42	0.80	1.00	0.23	1.00	0.53	0.19	0.56	1.00	0.34	0.52	0.20	0.18	0.19	0.12	0.31	0.06	0.40
5	0.25	0.36	0.85	0.39	1.12	0.50	0.16	0.19	2.45	0.90	0.30	0.42	0.44	0.19	0.15	0.05	0.05	0.10
6	0.29	0.42	1.61	0.30	1.57	0.14	0.14	0.23	2.45	0.21	0.50	0.25	0.23	0.14	0.13	0.25	0.05	0.24
7	0.20	0.57	0.95	0.19	2.45	0.85	0.21	0.33	1.14	0.32	0.42	0.22	0.24	0.23	0.10	0.18	0.14	0.38
8	0.46	0.79	0.73	0.27	1.00	1.17	0.34	0.20	1.59	0.38	0.34	0.28	0.26	0.10	0.15	0.33	0.05	0.66
9	1.31	0.35	0.97	0.42	1.12	1.56	0.29	0.56	2.45	0.73	0.61	0.29	0.52	0.13	0.23	0.55	0.03	0.33
10	0.36	0.32	1.41	0.12	2.45	1.15	0.22	0.31	2.45	0.45	1.10	0.26	0.31	0.57	0.06	0.19	0.08	0.39
11	1.82	0.36	0.68	0.26	0.95	2.45	0.45	0.97	2.45	0.57	0.34	0.30	0.23	0.24	0.11	0.62	0.10	0.43
12	1.19	0.35	1.00	0.21	0.90	0.64	0.32	0.72	1.00	0.42	0.19	0.33	0.13	0.42	0.12	0.15	0.07	0.41
mean	0.80	0.45	1.05	0.28	1.36	1.26	0.27	0.44	1.91	0.44	0.43	0.28	0.28	0.21	0.14	0.30	0.08	0.39
SD	0.70	0.18	0.43	0.09	0.54	0.78	0.11	0.26	0.66	0.19	0.23	0.06	0.13	0.15	0.04	0.18	0.04	0.13
CV	0.87	0.39	0.41	0.32	0.40	0.62	0.42	0.59	0.35	0.44	0.54	0.23	0.45	0.69	0.29	0.60	0.53	0.33

Subject	Lysophosphatidylcholins													
	lysoPCaC140	lysoPCaC160	lysoPCaC161	lysoPCaC170	lysoPCaC180	lysoPCaC181	lysoPCaC182	lysoPCaC203	lysoPCaC204	lysoPCaC240	lysoPCaC260	lysoPCaC261	lysoPCaC280	lysoPCaC281
1	0.17	0.08	0.16	0.18	0.22	0.36	0.17	0.36	0.24	0.36	0.45	0.34	0.34	1.75
2	0.08	0.11	0.10	0.06	0.12	0.14	0.22	0.40	0.28	0.67	0.38	0.48	0.42	0.31
3	0.05	0.08	0.08	0.05	0.06	0.09	0.09	0.24	0.30	0.25	0.55	0.25	0.44	0.34
4	0.04	0.08	0.09	0.11	0.07	0.08	0.15	0.32	0.24	0.40	0.61	0.34	0.38	0.32
5	0.03	0.15	0.12	0.09	0.05	0.14	0.16	0.47	0.22	0.93	0.43	0.41	0.87	0.28
6	0.11	0.06	0.13	0.10	0.10	0.16	0.18	0.24	0.32	0.42	0.34	0.40	0.24	0.38
7	0.10	0.18	0.09	0.15	0.15	0.26	0.17	0.40	0.21	0.27	0.34	0.21	0.27	0.59
8	0.07	0.08	0.10	0.07	0.07	0.10	0.08	0.36	0.25	0.17	0.40	0.25	0.30	0.47
9	0.10	0.28	0.05	0.09	0.10	0.27	0.20	0.26	0.30	0.37	0.41	0.26	0.42	1.19
10	0.11	0.17	0.06	0.11	0.17	0.33	0.11	0.24	0.34	0.27	0.50	0.38	0.46	0.38
11	0.10	0.11	0.12	0.16	0.10	0.16	0.25	0.30	0.25	0.34	0.56	0.23	0.36	0.36
12	0.10	0.11	0.07	0.11	0.18	0.29	0.14	0.46	0.19	0.63	0.70	0.22	0.33	1.10
mean	0.09	0.13	0.10	0.11	0.11	0.20	0.16	0.34	0.26	0.42	0.47	0.31	0.40	0.62
SD	0.03	0.06	0.03	0.04	0.05	0.09	0.05	0.08	0.04	0.21	0.11	0.08	0.15	0.45
CV	0.39	0.47	0.31	0.36	0.44	0.48	0.30	0.24	0.17	0.49	0.23	0.27	0.38	0.72

Subject	Diacyl-Phospatidylcholins																				
	PCaaC240	PCaaC260	PCaaC281	PCaaC300	PCaaC302	PCaaC320	PCaaC321	PCaaC322	PCaaC323	PCaaC341	PCaaC342	PCaaC343	PCaaC344	PCaaC360	PCaaC361	PCaaC362	PCaaC363	PCaaC364	PCaaC365	PCaaC366	PCaaC380
1	1.88	1.03	0.42	1.81	0.23	0.12	0.14	1.03	0.26	0.13	0.15	0.45	1.15	0.29	0.25	0.22	0.12	0.14	0.50	0.54	2.37
2	0.33	0.29	0.20	1.11	0.13	0.19	0.29	0.47	0.14	0.08	0.24	0.44	0.38	0.19	0.12	0.12	0.18	0.16	0.57	0.47	1.26
3	0.28	0.11	0.12	2.19	0.11	0.06	0.14	0.18	0.05	0.05	0.05	0.16	0.10	0.07	0.03	0.06	0.17	0.22	0.30	0.09	0.95
4	0.39	0.16	0.11	1.24	0.18	0.07	0.14	0.24	0.04	0.03	0.06	0.18	0.25	0.04	0.02	0.08	0.10	0.17	0.30	0.15	1.16
5	0.32	0.10	0.18	1.65	0.16	0.13	0.33	0.32	0.16	0.05	0.22	0.17	0.32	0.12	0.03	0.12	0.10	0.09	0.27	0.15	1.08
6	0.36	0.27	0.15	1.32	0.33	0.12	0.16	0.34	0.07	0.15	0.30	0.19	0.23	0.15	0.09	0.20	0.22	0.27	0.34	0.28	2.45
7	0.47	0.25	0.32	1.40	0.37	0.11	0.13	0.43	0.08	0.13	0.21	0.27	0.33	0.17	0.13	0.27	0.16	0.21	0.62	0.27	1.76
8	0.46	0.24	0.31	1.13	0.19	0.06	0.20	0.16	0.05	0.10	0.13	0.24	0.34	0.07	0.06	0.13	0.23	0.40	0.53	0.24	1.20
9	0.88	0.49	0.49	1.34	0.10	0.15	0.11	0.47	0.09	0.07	0.21	0.14	0.78	0.09	0.11	0.18	0.27	0.29	0.55	0.50	1.31
10	0.39	0.17	0.19	1.37	0.16	0.17	0.25	0.28	0.08	0.08	0.26	0.20	0.22	0.09	0.11	0.15	0.20	0.32	0.20	0.15	1.57
11	0.40	0.16	0.34	1.62	0.14	0.11	0.17	0.49	0.15	0.08	0.14	0.25	0.39	0.30	0.05	0.15	0.10	0.29	0.57	0.26	1.13
12	0.97	0.40	0.47	1.15	0.07	0.17	0.17	0.84	0.15	0.18	0.14	0.18	0.96	0.05	0.13	0.14	0.29	0.15	0.27	0.54	1.69
mean	0.60	0.31	0.28	1.44	0.18	0.12	0.19	0.44	0.11	0.09	0.18	0.24	0.45	0.14	0.09	0.15	0.18	0.23	0.42	0.30	1.49
SD	0.44	0.24	0.13	0.31	0.09	0.04	0.07	0.25	0.06	0.04	0.08	0.10	0.31	0.08	0.06	0.06	0.06	0.08	0.14	0.16	0.47
**CV**	**0.74**	**0.80**	**0.47**	**0.21**	**0.48**	**0.36**	**0.35**	**0.57**	**0.56**	**0.45**	**0.42**	**0.42**	**0.69**	**0.62**	**0.65**	**0.37**	**0.36**	**0.38**	**0.35**	**0.52**	**0.32**

Subject	Diacyl-Phospatidylcholins																
	PCaaC381	PCaaC383	PCaaC384	PCaaC385	PCaaC386	PCaaC401	PCaaC402	PCaaC403	PCaaC404	PCaaC405	PCaaC406	PCaaC420	PCaaC421	PCaaC422	PCaaC424	PCaaC425	PCaaC426
1	0.12	0.19	0.10	0.10	1.87	1.54	0.69	0.37	0.31	0.70	1.75	1.10	1.73	0.89	1.19	1.78	1.51
2	0.29	0.22	0.22	0.20	0.53	0.19	0.42	0.31	0.43	0.17	0.38	0.38	0.44	0.59	0.17	0.35	0.46
3	0.05	0.19	0.20	0.19	0.24	0.17	0.26	0.11	0.16	0.10	0.28	0.27	0.28	0.11	0.24	0.35	0.30
4	0.07	0.15	0.13	0.13	0.43	0.37	0.18	0.15	0.13	0.08	0.31	0.37	0.42	0.23	0.39	0.45	0.19
5	0.13	0.09	0.09	0.15	0.38	0.38	0.21	0.15	0.24	0.07	0.22	0.31	0.37	0.42	0.34	0.33	0.17
6	0.11	0.20	0.16	0.15	0.30	0.47	0.12	0.19	0.23	0.18	0.50	0.21	0.32	0.38	0.32	0.38	0.31
7	0.06	0.16	0.18	0.18	0.52	0.37	0.25	0.07	0.15	0.14	0.37	0.36	0.48	0.40	0.61	0.44	0.33

8	0.07	0.21	0.20	0.27	0.44	0.44	0.29	0.13	0.35	0.18	0.38	0.38	0.39	0.39	0.18	0.42	0.28
9	0.18	0.29	0.30	0.25	1.02	0.69	0.62	0.36	0.49	0.31	0.88	1.22	0.94	1.12	0.44	0.89	0.91
10	0.09	0.17	0.14	0.25	0.38	0.32	0.38	0.17	0.16	0.14	0.33	0.27	0.35	0.46	0.19	0.30	0.24
11	0.13	0.14	0.19	0.17	0.43	0.29	0.34	0.10	0.22	0.18	0.44	0.38	0.35	0.59	0.53	0.45	0.24
12	0.18	0.31	0.27	0.22	1.02	0.88	0.18	0.18	0.36	0.31	0.92	0.89	1.00	0.96	0.38	1.13	0.90
mean	0.12	0.19	0.18	0.19	0.63	0.51	0.33	0.19	0.27	0.21	0.56	0.51	0.59	0.54	0.42	0.61	0.49
SD	0.07	0.06	0.06	0.05	0.44	0.37	0.17	0.10	0.11	0.16	0.42	0.33	0.41	0.29	0.27	0.43	0.39
CV	0.54	0.30	0.33	0.27	0.71	0.72	0.51	0.50	0.42	0.77	0.74	0.65	0.70	0.53	0.65	0.71	0.80

Subject	Acyl-Alkyl-Phosphatidylcholins																				
	**PCaeC300**	**PCaeC301**	**PCaeC302**	**PCaeC321**	**PCaeC322**	**PCaeC340**	**PCaeC341**	**PCaeC342**	**PCaeC343**	**PCaeC360**	**PCaeC361**	**PCaeC362**	**PCaeC363**	**PCaeC364**	**PCaeC365**	**PCaeC380**	**PCaeC381**	**PCaeC382**	**PCaeC383**	**PCaeC384**	**PCaeC385**
1	1.04	1.21	0.34	1.51	0.23	0.25	0.25	0.17	0.93	0.14	0.17	0.37	0.14	0.15	1.28	0.26	0.13	0.13	0.06	0.13	0.21
2	0.84	0.42	0.29	0.33	0.16	0.09	0.34	0.32	0.28	0.10	0.13	0.29	0.21	0.13	0.32	0.58	0.38	0.25	0.26	0.24	0.57
3	0.21	0.23	0.19	0.32	0.08	0.18	0.17	0.08	0.09	0.11	0.18	0.31	0.16	0.13	0.22	0.27	0.23	0.13	0.20	0.09	0.11
4	0.40	0.38	0.19	0.26	0.25	0.18	0.11	0.08	0.26	0.09	0.14	0.24	0.16	0.08	0.20	0.65	0.39	0.15	0.20	0.17	0.14
5	0.81	0.29	0.25	0.33	0.18	0.29	0.33	0.29	0.13	0.20	0.37	0.48	0.27	0.13	0.19	0.71	0.60	0.55	0.26	0.23	0.20
6	0.69	0.13	0.24	0.25	0.31	0.29	0.23	0.11	0.30	0.28	0.30	0.33	0.24	0.12	0.20	0.91	0.43	0.30	0.24	0.23	0.14
7	0.54	0.28	0.43	0.43	0.35	0.31	0.30	0.28	0.32	0.17	0.21	0.23	0.16	0.20	0.31	0.59	0.19	0.13	0.16	0.20	0.15
8	0.73	0.32	0.12	0.35	0.14	0.27	0.21	0.08	0.23	0.23	0.29	0.32	0.18	0.18	0.28	0.37	0.39	0.39	0.19	0.16	0.25
9	0.63	0.61	0.18	0.73	0.26	0.18	0.21	0.07	0.64	0.30	0.14	0.15	0.31	0.10	0.86	0.78	0.59	0.22	0.12	0.22	0.31
10	1.12	0.18	0.27	0.29	0.19	0.26	0.28	0.22	0.20	0.18	0.31	0.35	0.14	0.14	0.18	0.51	0.44	0.14	0.13	0.17	0.16
11	0.59	0.39	0.35	0.31	0.20	0.37	0.29	0.29	0.23	0.29	0.27	0.36	0.39	0.40	0.37	0.64	0.26	0.27	0.31	0.23	0.19
12	1.43	0.92	0.57	0.78	0.21	0.10	0.29	0.34	0.83	0.26	0.09	0.26	0.18	0.23	0.84	0.87	0.21	0.12	0.14	0.15	0.25
mean	0.75	0.45	0.29	0.49	0.21	0.23	0.25	0.19	0.37	0.20	0.22	0.31	0.21	0.17	0.44	0.59	0.35	0.23	0.19	0.18	0.22
SD	0.32	0.31	0.12	0.35	0.07	0.08	0.07	0.10	0.26	0.07	0.08	0.08	0.07	0.08	0.34	0.20	0.15	0.13	0.07	0.05	0.12
CV	0.42	0.68	0.42	0.71	0.33	0.35	0.26	0.53	0.71	0.37	0.39	0.26	0.35	0.48	0.78	0.34	0.41	0.54	0.36	0.24	0.52

Subject	Acyl-Alkyl-Phosphatidylcholinss																
	**PCaeC386**	**PCaeC401**	**PCaeC402**	**PCaeC403**	**PCaeC404**	**PCaeC405**	**PCaeC406**	**PCaeC420**	**PCaeC421**	**PCaeC422**	**PCaeC423**	**PCaeC424**	**PCaeC425**	**PCaeC443**	**PCaeC444**	**PCaeC445**	**PCaeC446**
1	0.27	0.15	0.26	1.18	0.09	0.34	1.83	1.78	0.46	0.25	0.81	1.83	1.32	1.75	1.25	1.38	0.61
2	0.69	0.33	0.29	0.22	0.51	0.21	0.40	0.46	0.52	0.53	1.00	0.33	0.22	0.31	0.43	0.41	0.30
3	0.16	0.13	0.29	0.14	0.23	0.10	0.26	0.16	0.28	0.32	0.41	0.24	0.30	0.27	0.27	0.15	0.03
4	0.23	0.19	0.33	0.22	0.13	0.12	0.43	0.34	0.34	0.44	1.26	0.32	0.36	0.32	0.29	0.22	0.07
5	0.48	0.39	0.65	0.32	0.34	0.16	0.34	0.29	0.60	0.53	1.54	0.29	0.33	0.36	0.31	0.24	0.05
6	0.13	0.32	0.28	0.25	0.25	0.15	0.41	0.34	0.56	0.51	1.03	0.37	0.56	0.35	0.41	0.24	0.06
7	0.45	0.28	0.23	0.21	0.21	0.24	0.46	0.36	0.37	0.33	1.55	0.35	0.56	0.33	0.14	0.41	0.14
8	0.36	0.38	0.52	0.24	0.24	0.15	0.44	0.45	0.60	0.55	1.19	0.41	0.30	0.32	0.16	0.13	0.03
9	0.68	0.24	0.29	0.54	0.10	0.05	0.93	0.85	0.22	0.30	2.45	0.89	0.94	0.98	0.70	0.31	0.37
10	0.37	0.26	0.45	0.31	0.31	0.09	0.35	0.30	0.50	0.54	1.25	0.30	0.29	0.26	0.30	0.22	0.09
11	0.34	0.21	0.50	0.18	0.38	0.31	0.39	0.44	0.59	0.59	2.45	0.35	0.52	0.31	0.21	0.22	0.12
12	0.43	0.31	0.21	0.62	0.15	0.26	0.95	0.95	0.71	0.70	1.00	0.85	1.04	0.91	0.84	0.77	0.13
mean	0.38	0.26	0.36	0.37	0.24	0.18	0.60	0.56	0.48	0.47	1.33	0.54	0.56	0.54	0.44	0.39	0.17
SD	0.17	0.08	0.13	0.28	0.12	0.09	0.43	0.43	0.14	0.13	0.58	0.44	0.34	0.43	0.32	0.34	0.17
CV	0.44	0.30	0.37	0.76	0.49	0.48	0.71	0.77	0.30	0.28	0.44	0.80	0.60	0.80	0.72	0.87	1.02

Subject	Sphingomyelins														
	**SMOHC141**	**SMOHC161**	**SMOHC221**	**SMOHC222**	**SMOHC241**	**SMC160**	**SMC161**	**SMC180**	**SMC181**	**SMC202**	**SMC223**	**SMC240**	**SMC241**	**SMC260**	**SMC261**
1	0.07	0.09	0.11	0.14	0.05	0.39	0.04	0.19	0.57	1.86	0.08	0.11	0.17	0.29	0.48
2	0.32	0.14	0.25	0.16	0.04	0.27	0.13	0.17	1.49	0.35	0.10	0.10	0.30	0.45	0.43
3	0.07	0.11	0.17	0.14	0.01	0.07	0.13	0.15	0.34	0.59	0.08	0.06	0.27	0.15	0.37
4	0.05	0.09	0.15	0.07	0.02	0.12	0.02	0.19	0.55	0.76	0.05	0.06	0.18	0.12	0.40
5	0.10	0.18	0.21	0.22	0.07	0.19	0.07	0.27	0.51	0.86	0.14	0.17	0.14	0.24	0.10
6	0.11	0.08	0.14	0.18	0.03	0.16	0.07	0.18	1.06	0.63	0.09	0.11	0.18	0.23	0.24
7	0.12	0.08	0.32	0.04	0.01	0.16	0.06	0.30	0.82	0.65	0.06	0.08	0.29	0.15	0.38
8	0.06	0.11	0.23	0.27	0.03	0.11	0.09	0.22	0.51	0.73	0.10	0.12	0.27	0.12	0.66
9	0.15	0.19	0.28	0.22	0.02	0.14	0.08	0.33	0.59	0.71	0.04	0.11	0.28	0.30	0.33
10	0.09	0.14	0.24	0.14	0.03	0.15	0.09	0.25	0.62	0.77	0.09	0.15	0.35	0.34	0.39
11	0.09	0.14	0.19	0.05	0.04	0.13	0.06	0.11	0.59	0.74	0.13	0.05	0.21	0.18	0.43
12	0.14	0.13	0.11	0.18	0.04	0.08	0.08	0.27	0.37	0.90	0.13	0.12	0.45	0.27	0.41
mean	0.11	0.12	0.20	0.15	0.03	0.16	0.08	0.22	0.67	0.80	0.09	0.10	0.26	0.24	0.39
SD	0.07	0.03	0.06	0.07	0.02	0.09	0.03	0.06	0.31	0.35	0.03	0.04	0.08	0.10	0.13
CV	0.60	0.27	0.32	0.45	0.47	0.53	0.39	0.29	0.46	0.44	0.35	0.34	0.32	0.41	0.33
