# Supplementary material for: Combined Targeted Analysis of Metabolites and Proteins in Tear Fluid With Regard to Clinical Applications
Source: Transl Vis Sci Technol. 2018 Dec 6;7(6):22. doi: 10.1167/tvst.7.6.22 (PMC6284467; doi:10.1167/tvst.7.6.22)
Supplement: Supplement 3 [file tvst-07-06-18_s03.pdf]

Title: Combined Targeted Analysis of Metabolites and Proteins in Tear Fluid with Regard to Clinical Applications

Jounal: TVST

Authors: Sascha Dammeier, Peter Martus, Franziska Klose, Michael Seid, Dario Bosch, Janina D‘Alvise, Focke Ziemssen, Spyridon Dimopoulos and Marius Ueffing

Corresponding Author:

Sascha Dammeier, Institute for Ophthalmic Research, Core Facility for Medical Bioanalytics, University of Tübingen, Elfriede-Aulhorn-Strasse 7, 72076 Tübingen, Germany, email: sascha.dammeier@uni-tuebingen.de

Supplementary Table S3. Comparison of shotgun proteomics of tear fluid with and without metabolomic sample preparation. Tear fluid has been taken by Schirmer-like strips. The punches have been processed for untargeted proteomic analysis either directly (Px only) or after metabolomic sample preparation (Mx+Px).

Subject	Eye	Sample Type	MS1 Features	MS/MS Features	MS/MS Submitted	MS/MS Identified	MS/MS Identified [%]	Protein IDs *(LFQ intensity)*
1	right	Px only	4596	20068	21779	7079	32.5	653
1	right	Px only	4674	20013	21691	7084	32.66	658
15	right	Px only	4598	17680	19114	5028	26.31	542
1	right	Mx+Px	4975	20177	21641	4123	19.05	692
3	right	Mx+Px	4871	19563	20866	4585	21.97	700
4	right	Mx+Px	5016	20080	21572	4206	19.5	706
