# Supplementary material for: Combined Targeted Analysis of Metabolites and Proteins in Tear Fluid With Regard to Clinical Applications
Source: Transl Vis Sci Technol. 2018 Dec 6;7(6):22. doi: 10.1167/tvst.7.6.22 (PMC6284467; doi:10.1167/tvst.7.6.22)
Supplement: Supplement 4 [file tvst-07-06-18_s04.pdf]

Title: Combined Targeted Analysis of Metabolites and Proteins in Tear Fluid with Regard to Clinical Applications
Journal: TVST
Authors: Sascha Dammeier, Peter Martus, Franziska Klose, Michael Seid, Dario Bosch, Janina D‘Alvise, Focke Ziemssen, Spyridon Dimopoulos and Marius Ueffing
Corresponding Author: Sascha Dammeier, Institute for Ophthalmic Research, Core Facility for Medical Bioanalytics, University of Tübingen, Elfriede-Aulhorn-Strasse 7, 72076 Tübingen, Germany, email: sascha.dammeier@uni-tuebingen.de

Supplementary Table S4. Intra-individual variations of all analytes according to subjects with regard to the donating eye. Coefficients of variation were calculated from normalized concentration values of each subject. (3 tear fluid donations per left eye (L) and per right eye (R)). Red numbers indicate imputed values.

Subject	Eye	Amino Acids																				
		**Ala**	**Arg**	**Asn**	**Asp**	**Cit**	**Gln**	**Glu**	**Gly**	**His**	**Ile**	**Leu**	**Lys**	**Met**	**Orn**	**Phe**	**Pro**	**Ser**	**Thr**	**Trp**	**Tyr**	**Val**
1	L	0.11	0.37	0.21	0.29	0.13	0.13	0.12	0.22	0.55	0.58	0.29	0.09	0.26	0.30	0.27	0.13	0.02	0.25	0.22	0.41	0.45
	R	0.07	0.16	0.08	0.46	0.15	0.06	0.23	0.08	0.06	0.09	0.10	0.12	0.13	0.07	0.20	0.09	0.07	0.01	0.10	0.10	0.23
2	L	0.10	0.06	0.51	0.05	1.08	0.04	0.08	0.16	0.14	1.73	0.39	0.46	0.18	0.17	0.18	0.16	0.13	0.40	0.30	0.14	0.44
	R	0.16	0.23	0.23	0.08	0.97	0.16	0.17	0.14	0.44	1.13	0.21	0.24	0.39	0.13	0.06	0.14	0.11	0.22	0.21	0.19	0.21
3	L	0.13	0.28	0.25	0.22	0.47	0.10	0.13	0.26	0.29	0.29	0.33	0.19	0.50	0.20	0.19	0.25	0.21	0.16	0.27	0.30	0.88
	R	0.05	0.15	0.09	0.37	0.14	0.06	0.15	0.11	0.21	0.25	0.20	0.19	0.30	0.09	0.08	0.15	0.10	0.15	0.13	0.21	0.07
4	L	0.08	0.30	0.12	0.10	0.34	0.15	0.08	0.09	0.15	0.21	0.12	0.07	0.19	0.25	0.10	0.10	0.09	0.14	0.04	0.02	0.25
	R	0.10	0.04	0.29	0.07	0.10	0.05	0.20	0.08	0.35	0.26	0.08	0.12	0.25	0.02	0.07	0.07	0.12	0.04	0.22	0.30	0.90
5	L	0.13	0.13	0.06	0.52	0.66	0.08	0.20	0.14	0.17	0.21	0.13	0.10	0.34	0.19	0.04	0.16	0.21	0.15	0.09	0.09	0.30
	R	0.23	0.49	0.26	0.09	0.49	0.32	0.18	0.28	0.17	0.59	0.39	0.21	0.15	0.56	0.46	0.26	0.24	0.62	0.14	0.22	0.38
6	L	0.03	0.28	0.06	0.13	0.11	0.08	0.14	0.15	0.11	0.18	0.17	0.17	0.18	0.26	0.13	0.25	0.07	0.20	0.06	0.08	0.16
	R	0.09	0.15	0.02	0.10	0.02	0.07	0.15	0.01	0.14	0.23	0.11	0.08	0.18	0.13	0.06	0.10	0.09	0.08	0.10	0.08	0.31
7	L	0.05	0.09	0.01	0.18	0.26	0.14	0.22	0.04	0.30	0.27	0.07	0.14	0.32	0.08	0.07	0.02	0.13	0.13	0.17	0.15	0.21
	R	0.09	0.10	0.13	0.18	0.22	0.13	0.08	0.09	0.18	0.16	0.09	0.15	0.26	0.09	0.05	0.12	0.18	0.09	0.25	0.19	1.09
8	L	0.07	0.23	0.30	0.20	0.25	0.07	0.09	0.05	0.07	0.13	0.10	0.07	0.17	0.23	0.15	0.14	0.09	0.29	0.12	0.07	0.12
	R	0.17	0.37	0.17	0.14	0.60	0.15	0.04	0.16	0.28	0.26	0.42	0.10	0.27	0.24	0.17	0.12	0.12	0.34	0.13	0.33	0.31
9	L	0.20	0.25	0.10	0.43	0.27	0.04	0.09	0.18	0.14	0.12	0.18	0.31	0.25	0.29	0.31	0.10	0.23	0.28	0.28	0.15	0.29
	R	0.15	0.62	0.05	0.25	1.05	0.18	0.03	0.48	0.20	0.50	0.04	0.06	0.65	0.31	0.17	0.64	0.20	0.11	0.18	0.13	0.11
10	L	0.05	0.27	0.04	0.14	0.13	0.01	0.10	0.11	0.07	0.07	0.07	0.11	0.04	0.22	0.18	0.23	0.12	0.14	0.05	0.14	0.24
	R	0.07	0.13	0.02	0.25	0.30	0.06	0.10	0.06	0.15	0.05	0.20	0.10	0.10	0.07	0.14	0.14	0.10	0.04	0.09	0.22	0.27
11	L	0.08	0.10	0.11	0.20	0.36	0.04	0.22	0.20	0.28	0.59	0.08	0.09	0.22	0.14	0.17	0.09	0.13	0.04	0.13	0.37	0.27
	R	0.12	0.13	0.18	0.07	0.42	0.06	0.04	0.07	0.18	0.39	0.15	0.12	0.32	0.05	0.39	0.06	0.09	0.10	0.07	0.10	0.88
12	L	0.09	0.13	0.07	0.09	0.16	0.06	0.12	0.12	0.15	0.12	0.05	0.17	0.10	0.11	0.12	0.09	0.19	0.06	0.11	0.15	0.07
	R	0.23	0.29	0.11	0.44	0.67	0.19	0.22	0.54	0.30	0.34	0.26	0.27	1.17	0.23	0.24	0.69	0.31	0.09	0.10	0.10	0.88
mean		0.11	0.22	0.14	0.21	0.39	0.10	0.13	0.16	0.21	0.36	0.18	0.16	0.29	0.18	0.17	0.18	0.14	0.17	0.15	0.18	0.39
SD		0.05	0.14	0.12	0.14	0.30	0.07	0.06	0.12	0.12	0.37	0.11	0.09	0.23	0.11	0.10	0.16	0.07	0.14	0.07	0.10	0.29
CV		0.49	0.62	0.79	0.64	0.77	0.66	0.46	0.78	0.55	1.01	0.64	0.58	0.78	0.62	0.63	0.88	0.48	0.79	0.50	0.56	0.76

Subject	Eye	Acylcarnitines																				
		**C0**	**C10**	**C101**	**C102**	**C12**	**C12DC**	**C121**	**C14**	**C141**	**C141OH**	**C142**	**C142OH**	**C16**	**C16OH**	**C161**	**C161OH**	**C162**	**C162OH**	**C18**	**C181**	**C181OH**
1	L	0.25	0.25	0.46	0.35	0.37	0.36	0.31	0.41	0.48	0.57	0.41	0.08	0.31	0.37	0.40	0.44	0.40	0.25	0.38	0.33	0.20
	R	0.09	0.08	0.06	0.02	0.11	0.03	0.05	0.15	0.12	0.14	0.15	0.18	0.07	0.10	0.12	0.24	0.02	0.08	0.31	0.29	0.07
2	L	0.33	0.37	0.35	0.41	0.41	0.37	0.34	0.30	0.43	0.44	0.41	0.28	0.29	0.29	0.44	0.50	0.30	0.45	0.34	0.34	0.42
	R	0.27	0.18	0.17	0.26	0.28	0.14	0.18	0.22	0.23	0.34	0.28	0.19	0.23	0.14	0.32	0.30	0.15	0.30	0.28	0.28	0.08
3	L	0.02	0.12	0.07	0.23	0.08	0.07	0.11	0.20	0.07	0.00	0.09	0.01	0.09	0.03	0.02	0.14	0.20	0.13	0.06	0.19	0.32
	R	0.12	0.09	0.14	0.07	0.21	0.16	0.15	0.09	0.25	0.26	0.17	0.14	0.18	0.25	0.30	0.19	0.21	0.14	0.12	0.29	0.07
4	L	0.18	0.23	0.15	0.26	0.25	0.17	0.16	0.14	0.11	0.26	0.19	0.06	0.21	0.19	0.17	0.20	0.33	0.18	0.11	0.19	0.04
	R	0.16	0.19	0.18	0.12	0.12	0.10	0.10	0.25	0.17	0.25	0.13	0.15	0.13	0.14	0.27	0.24	0.13	0.38	0.05	0.04	0.09
5	L	0.13	0.28	0.15	0.26	0.07	0.23	0.19	0.26	0.24	0.02	0.10	0.25	0.13	0.17	0.35	0.16	0.21	0.20	0.48	0.40	0.23
	R	0.30	0.55	0.34	0.35	0.24	0.39	0.43	0.43	0.38	0.33	0.33	0.24	0.40	0.24	0.37	0.27	0.34	0.23	0.95	0.95	0.19
6	L	0.13	0.09	0.07	0.07	0.10	0.11	0.17	0.11	0.04	0.17	0.14	0.06	0.11	0.19	0.34	0.13	0.39	0.06	0.27	0.26	0.13
	R	0.09	0.08	0.05	0.12	0.08	0.11	0.07	0.02	0.07	0.07	0.09	0.12	0.10	0.06	0.18	0.10	0.11	0.09	0.06	0.11	0.13
7	L	0.31	0.31	0.29	0.26	0.29	0.37	0.22	0.36	0.37	0.41	0.30	0.20	0.41	0.19	0.27	0.28	0.26	0.28	0.75	0.80	0.09
	R	0.04	0.20	0.02	0.21	0.13	0.19	0.09	0.10	0.13	0.16	0.12	0.05	0.09	0.14	0.06	0.11	0.06	0.23	0.22	0.20	0.01
8	L	0.10	0.10	0.20	0.11	0.23	0.12	0.10	0.09	0.15	0.17	0.21	0.21	0.30	0.15	0.23	0.33	0.32	0.10	0.21	0.25	0.14
	R	0.17	0.20	0.22	0.11	0.17	0.22	0.19	0.12	0.08	0.32	0.11	0.25	0.14	0.14	0.05	0.30	0.22	0.23	0.14	0.14	0.09
9	L	0.24	0.15	0.22	0.17	0.32	0.18	0.26	0.31	0.32	0.25	0.23	0.27	0.30	0.32	0.20	0.18	0.44	0.27	0.27	0.21	0.21
	R	0.21	0.36	0.16	0.26	0.12	0.27	0.15	0.24	0.25	0.12	0.11	0.16	0.16	0.23	0.04	0.14	0.02	0.06	0.11	0.08	0.20
10	L	0.22	0.24	0.16	0.11	0.21	0.13	0.13	0.19	0.12	0.19	0.22	0.09	0.10	0.11	0.13	0.26	0.30	0.11	0.28	0.23	0.17
	R	0.03	0.08	0.07	0.09	0.06	0.13	0.09	0.12	0.05	0.02	0.10	0.10	0.06	0.04	0.16	0.08	0.04	0.17	0.22	0.08	0.19
11	L	0.16	0.13	0.19	0.03	0.10	0.14	0.10	0.18	0.18	0.11	0.14	0.09	0.10	0.24	0.04	0.20	0.05	0.16	0.10	0.13	0.09
	R	0.04	0.11	0.12	0.13	0.05	0.05	0.12	0.11	0.13	0.09	0.12	0.14	0.23	0.09	0.06	0.09	0.24	0.02	0.57	0.46	0.12
12	L	0.12	0.33	0.08	0.21	0.28	0.30	0.08	0.20	0.08	0.16	0.30	0.25	0.32	0.16	0.34	0.34	0.27	0.30	0.25	0.27	0.24
	R	0.40	0.37	0.27	0.47	0.24	0.34	0.42	0.44	0.22	0.26	0.60	0.26	0.27	0.47	0.46	0.36	0.27	0.60	0.16	0.22	0.35
mean		0.17	0.21	0.17	0.19	0.19	0.19	0.18	0.21	0.20	0.21	0.21	0.16	0.20	0.18	0.22	0.23	0.22	0.21	0.28	0.28	0.16

SD		0.10	0.12	0.11	0.12	0.10	0.11	0.10	0.11	0.12	0.14	0.12	0.08	0.10	0.10	0.13	0.11	0.12	0.13	0.22	0.21	0.10
CV		0.59	0.56	0.61	0.61	0.54	0.55	0.59	0.54	0.63	0.65	0.59	0.49	0.53	0.56	0.61	0.47	0.56	0.63	0.77	0.73	0.61

Subject	Eye	Acylcarnitines																		
		**C182**	**C2**	**C3**	**C3DCC4OH**	**C3OH**	**C31**	**C4**	**C41**	**C6C41DC**	**C5**	**C5MDC**	**C5OHC3DCM**	**C51**	**C51DC**	**C5DCC6OH**	**C61**	**C7DC**	**C8**	**C9**
1	L	0.18	0.15	0.21	0.36	0.50	0.10	0.42	0.23	0.23	0.35	0.12	0.35	0.37	0.43	0.33	0.37	0.38	0.29	0.57
	R	0.17	0.14	0.03	0.14	0.18	0.06	0.21	0.10	0.22	0.12	0.21	0.02	0.02	0.15	0.16	0.12	0.10	0.04	0.18
2	L	0.20	0.32	0.33	0.31	0.29	0.15	0.37	0.25	0.31	0.55	0.63	0.33	0.41	0.32	0.32	0.36	0.30	0.39	0.34
	R	0.19	0.15	0.29	0.23	0.12	0.19	0.30	0.23	0.16	0.24	0.26	0.22	0.27	0.23	0.15	0.10	0.26	0.31	0.24
3	L	0.03	0.07	0.13	0.06	0.13	0.03	0.15	0.05	0.17	0.46	0.69	0.04	0.21	0.13	0.18	0.29	0.17	0.10	0.24
	R	0.06	0.10	0.06	0.14	0.02	0.13	0.11	0.08	0.18	0.58	0.78	0.19	0.26	0.26	0.09	0.12	0.16	0.16	0.43
4	L	0.14	0.10	0.19	0.19	0.18	0.05	0.14	0.15	0.12	0.44	0.58	0.11	0.26	0.18	0.20	0.32	0.16	0.24	0.46
	R	0.09	0.06	0.08	0.22	0.23	0.08	0.18	0.17	0.23	0.43	0.50	0.11	0.26	0.16	0.20	0.15	0.07	0.26	0.16
5	L	0.09	0.04	0.11	0.03	0.17	0.21	0.06	0.15	0.17	0.18	0.21	0.12	0.14	0.13	0.13	0.09	0.18	0.12	0.38
	R	0.02	0.12	0.10	0.14	0.14	0.14	0.17	0.29	0.18	0.49	0.60	0.13	0.38	0.16	0.17	0.22	0.34	0.60	0.26
6	L	0.28	0.20	0.14	0.19	0.16	0.18	0.08	0.14	0.07	0.31	0.60	0.16	0.13	0.15	0.18	0.39	0.24	0.16	0.15
	R	0.04	0.15	0.09	0.14	0.13	0.05	0.06	0.13	0.00	0.39	0.22	0.04	0.15	0.07	0.02	0.17	0.06	0.17	0.36
7	L	0.27	0.24	0.31	0.24	0.31	0.05	0.45	0.34	0.63	0.57	0.60	0.36	0.37	0.37	0.27	0.34	0.23	0.36	0.41
	R	0.11	0.25	0.10	0.06	0.15	0.12	0.16	0.08	0.19	0.56	0.68	0.12	0.15	0.10	0.12	0.26	0.22	0.09	0.05
8	L	0.11	0.12	0.07	0.18	0.24	0.09	0.18	0.09	0.09	0.46	0.49	0.11	0.17	0.19	0.18	0.12	0.26	0.10	0.35
	R	0.16	0.25	0.08	0.08	0.10	0.05	0.11	0.12	0.19	0.66	0.84	0.12	0.29	0.15	0.09	0.08	0.13	0.23	0.28
9	L	0.28	0.37	0.26	0.16	0.34	0.13	0.25	0.18	0.03	0.24	0.12	0.27	0.16	0.27	0.26	0.27	0.36	0.25	0.28
	R	0.13	0.25	0.09	0.21	0.09	0.10	0.04	0.14	0.17	0.47	0.71	0.03	0.26	0.16	0.19	0.25	0.23	0.11	0.38
10	L	0.03	0.20	0.08	0.22	0.18	0.18	0.28	0.05	0.34	0.51	0.57	0.16	0.27	0.20	0.27	0.21	0.20	0.24	0.38
	R	0.10	0.35	0.10	0.07	0.08	0.04	0.07	0.08	0.35	0.08	0.14	0.08	0.09	0.01	0.12	0.23	0.07	0.04	0.40
11	L	0.08	0.22	0.07	0.21	0.14	0.12	0.11	0.13	0.28	0.46	0.64	0.12	0.24	0.15	0.25	0.29	0.30	0.14	0.23
	R	0.08	0.43	0.08	0.13	0.15	0.12	0.11	0.14	0.71	0.31	0.59	0.11	0.06	0.11	0.09	0.08	0.09	0.02	0.31
12	L	0.09	0.20	0.16	0.13	0.08	0.09	0.11	0.10	0.18	0.21	0.51	0.14	0.08	0.07	0.10	0.34	0.20	0.11	0.33
	R	0.15	0.32	0.23	0.37	0.39	0.07	0.33	0.35	0.12	0.03	0.32	0.27	0.18	0.31	0.41	0.55	0.43	0.33	0.23
mean		0.13	0.20	0.14	0.18	0.19	0.11	0.19	0.16	0.22	0.38	0.48	0.15	0.22	0.19	0.19	0.24	0.21	0.20	0.31
SD		0.07	0.10	0.08	0.09	0.11	0.05	0.12	0.08	0.16	0.17	0.22	0.10	0.10	0.10	0.09	0.12	0.10	0.13	0.11
CV		0.58	0.51	0.60	0.50	0.58	0.48	0.63	0.52	0.72	0.44	0.45	0.62	0.48	0.52	0.48	0.49	0.47	0.66	0.36

Subject	Eye	Biogenic Amines																Sugars
		**AcOrn**	**ADMA**	**c4OHPro**	**Carnosine**	**Creatinine**	**Dopamine**	**Histamine**	**Kynurenine**	**PEA**	**Putrescine**	**Serotonin**	**Spermidine**	**Spermine**	**t4OHPro**	**Taurine**	**SDMA**	**Hexose**
1	L	0.87	0.87	0.50	0.31	0.87	0.52	0.35	0.29	0.32	0.29	0.26	0.26	0.02	0.22	0.09	0.11	0.73
	R	0.09	0.24	0.26	0.17	1.73	1.00	0.15	0.07	0.19	0.10	0.09	0.03	0.04	0.10	0.20	0.08	0.07
2	L	1.73	0.57	1.00	0.25	1.73	1.73	0.50	1.73	0.24	0.46	0.40	0.62	0.25	0.03	0.26	0.22	0.66
	R	1.00	0.20	1.00	0.53	1.73	1.00	0.27	0.06	0.41	0.00	0.28	0.24	0.16	0.19	0.87	0.20	0.17
3	L	0.57	0.21	1.73	0.21	1.00	1.00	0.03	0.11	0.38	0.59	0.21	0.07	0.07	0.17	0.18	0.04	0.50
	R	0.12	0.26	1.73	0.23	1.00	1.73	0.04	0.36	0.20	0.09	0.09	0.18	0.05	0.12	0.32	0.08	0.25
4	L	0.29	0.88	1.00	0.27	1.00	0.16	0.14	0.57	0.55	0.75	0.11	0.08	0.13	0.05	0.37	0.06	0.17
	R	0.55	0.87	1.00	0.19	1.00	0.90	0.21	0.67	0.09	0.38	0.15	0.19	0.20	0.13	0.25	0.07	0.63
5	L	0.13	0.47	0.96	0.13	1.73	0.87	0.16	0.14	0.84	0.38	0.40	0.48	0.01	0.21	0.05	0.05	0.08
	R	0.37	0.31	0.87	0.55	0.88	0.15	0.19	0.15	0.33	0.28	0.13	0.21	0.30	0.00	0.06	0.06	0.13
6	L	0.32	0.14	1.00	0.32	1.73	0.14	0.13	0.19	0.23	0.68	0.15	0.24	0.10	0.19	0.39	0.06	0.35
	R	0.17	0.60	0.93	0.32	1.73	0.18	0.16	0.26	0.25	0.21	0.15	0.20	0.15	0.08	0.08	0.01	0.13
7	L	0.16	0.19	1.07	0.05	1.73	0.28	0.24	0.24	0.25	0.46	0.16	0.15	0.29	0.06	0.26	0.14	0.57
	R	0.25	0.89	1.03	0.22	1.00	1.73	0.23	0.30	0.41	0.35	0.30	0.35	0.22	0.02	0.10	0.05	0.19
8	L	0.37	0.13	0.68	0.16	1.00	1.73	0.22	0.27	0.43	0.23	0.35	0.30	0.14	0.06	0.38	0.03	0.58
	R	0.23	1.73	0.93	0.36	1.00	0.89	0.32	0.07	0.38	0.45	0.22	0.23	0.02	0.21	0.31	0.07	0.73
9	L	1.73	0.40	1.06	0.12	1.00	0.88	0.44	0.21	0.73	0.73	0.30	0.60	0.17	0.18	0.20	0.04	0.37
	R	0.86	0.35	1.01	0.56	0.20	1.00	0.15	0.90	0.28	0.34	0.06	0.35	0.10	0.32	0.73	0.03	0.35
10	L	0.36	0.41	1.46	0.10	1.73	1.00	0.26	0.31	0.67	1.03	0.20	0.05	0.66	0.07	0.18	0.07	0.32
	R	0.42	0.19	1.70	0.13	1.00	0.27	0.21	0.37	0.29	0.73	0.25	0.28	0.63	0.07	0.22	0.02	0.30
11	L	1.15	0.13	0.51	0.27	0.88	1.00	0.15	0.77	0.68	0.06	0.42	0.33	0.23	0.06	0.45	0.07	0.33
	R	1.00	0.55	0.71	0.21	0.89	1.73	0.16	0.67	0.37	0.52	0.21	0.10	0.28	0.11	0.44	0.12	0.54
12	L	1.73	0.15	1.00	0.19	1.73	0.26	0.21	0.62	0.35	0.09	0.25	0.06	0.43	0.02	0.15	0.10	0.51
	R	0.87	0.44	1.00	0.16	0.44	1.03	0.40	0.46	0.45	0.18	0.27	0.08	0.50	0.18	0.18	0.04	0.39
mean		0.64	0.47	1.01	0.25	1.20	0.88	0.22	0.41	0.39	0.39	0.23	0.24	0.22	0.12	0.28	0.08	0.38
SD		0.51	0.36	0.36	0.13	0.45	0.54	0.11	0.36	0.18	0.26	0.10	0.16	0.18	0.08	0.19	0.05	0.20
CV		0.81	0.78	0.35	0.53	0.38	0.62	0.50	0.88	0.47	0.65	0.45	0.67	0.82	0.66	0.69	0.68	0.54

Subject	Eye	Lysophosphatidylcholins													
		**lysoPCaC140**	**lysoPCaC160**	**lysoPCaC161**	**lysoPCaC170**	**lysoPCaC180**	**lysoPCaC181**	**lysoPCaC182**	**lysoPCaC203**	**lysoPCaC204**	**lysoPCaC240**	**lysoPCaC260**	**lysoPCaC261**	**lysoPCaC280**	**lysoPCaC281**
1	L	0.17	0.08	0.23	0.22	0.31	0.56	0.14	0.46	0.15	0.22	0.44	0.34	0.28	1.42
	R	0.08	0.10	0.08	0.02	0.02	0.12	0.23	0.14	0.17	0.37	0.25	0.24	0.28	0.22

Subject	Eye														
2	L	0.07	0.10	0.15	0.05	0.16	0.13	0.25	0.11	0.34	0.75	0.10	0.61	0.40	0.29
	R	0.10	0.13	0.06	0.06	0.08	0.16	0.24	0.44	0.20	0.19	0.62	0.42	0.36	0.40
3	L	0.04	0.08	0.13	0.02	0.06	0.07	0.12	0.32	0.28	0.19	0.32	0.26	0.14	0.23
	R	0.07	0.09	0.00	0.07	0.06	0.12	0.09	0.15	0.35	0.35	0.71	0.28	0.47	0.20
4	L	0.05	0.11	0.07	0.10	0.09	0.05	0.18	0.30	0.30	0.68	0.66	0.48	0.40	0.09
	R	0.02	0.02	0.12	0.11	0.06	0.11	0.13	0.41	0.22	0.03	0.67	0.15	0.39	0.42
5	L	0.02	0.16	0.14	0.13	0.06	0.17	0.20	0.51	0.19	0.97	0.47	0.44	1.02	0.14
	R	0.01	0.17	0.11	0.06	0.06	0.14	0.16	0.49	0.11	0.69	0.42	0.27	0.39	0.43
6	L	0.11	0.05	0.18	0.10	0.15	0.18	0.21	0.21	0.47	0.24	0.41	0.20	0.35	0.56
	R	0.07	0.08	0.04	0.03	0.04	0.11	0.16	0.17	0.20	0.62	0.34	0.59	0.15	0.26
7	L	0.14	0.23	0.11	0.24	0.21	0.38	0.26	0.64	0.13	0.42	0.49	0.25	0.26	0.67
	R	0.05	0.14	0.07	0.02	0.07	0.09	0.07	0.09	0.31	0.08	0.19	0.15	0.05	0.31
8	L	0.07	0.05	0.12	0.04	0.03	0.15	0.12	0.45	0.33	0.22	0.27	0.34	0.36	0.51
	R	0.07	0.04	0.08	0.06	0.09	0.05	0.03	0.16	0.17	0.01	0.34	0.17	0.24	0.29
9	L	0.03	0.09	0.04	0.11	0.05	0.11	0.15	0.15	0.34	0.40	0.27	0.22	0.32	0.04
	R	0.15	0.41	0.03	0.08	0.15	0.42	0.23	0.39	0.31	0.38	0.60	0.35	0.59	1.01
10	L	0.02	0.24	0.08	0.05	0.07	0.14	0.09	0.09	0.29	0.36	0.26	0.10	0.42	0.61
	R	0.16	0.09	0.05	0.07	0.17	0.41	0.11	0.37	0.30	0.19	0.76	0.63	0.55	0.19
11	L	0.10	0.12	0.11	0.10	0.01	0.10	0.21	0.33	0.29	0.35	0.50	0.17	0.05	0.10
	R	0.11	0.13	0.14	0.22	0.16	0.23	0.13	0.25	0.28	0.27	0.70	0.23	0.40	0.47
12	L	0.04	0.14	0.08	0.04	0.18	0.24	0.05	0.37	0.12	0.56	0.63	0.32	0.34	0.34
	R	0.10	0.07	0.03	0.17	0.17	0.37	0.17	0.51	0.22	0.26	0.41	0.06	0.31	1.16
mean		0.08	0.12	0.09	0.09	0.10	0.19	0.16	0.31	0.25	0.37	0.45	0.30	0.36	0.43
SD		0.05	0.08	0.05	0.06	0.07	0.13	0.06	0.16	0.09	0.23	0.18	0.15	0.19	0.34
CV		0.59	0.66	0.56	0.69	0.68	0.69	0.41	0.50	0.34	0.64	0.40	0.50	0.54	0.78

Subject	Eye	Diacyl-Phosphatidylcholins																			
		**PCaaC240**	**PCaaC260**	**PCaaC281**	**PCaaC300**	**PCaaC302**	**PCaaC320**	**PCaaC321**	**PCaaC322**	**PCaaC323**	**PCaaC341**	**PCaaC342**	**PCaaC343**	**PCaaC344**	**PCaaC360**	**PCaaC361**	**PCaaC362**	**PCaaC363**	**PCaaC364**	**PCaaC365**	**PCaaC366**	**PCaaC380**
1	L	1.52	1.05	0.58	1.29	0.31	0.18	0.18	1.00	0.40	0.21	0.22	0.48	1.14	0.41	0.40	0.34	0.19	0.13	0.83	0.68	1.73
	R	0.14	0.05	0.05	1.73	0.04	0.02	0.12	0.23	0.04	0.05	0.09	0.12	0.26	0.06	0.03	0.05	0.05	0.14	0.21	0.08	1.73
2	L	0.40	0.46	0.18	0.87	0.14	0.24	0.43	0.28	0.20	0.12	0.34	0.05	0.52	0.29	0.18	0.18	0.23	0.12	0.45	0.51	0.91
	R	0.16	0.13	0.20	1.73	0.13	0.17	0.18	0.57	0.05	0.02	0.13	0.76	0.08	0.01	0.04	0.05	0.13	0.23	0.73	0.23	1.73
3	L	0.27	0.12	0.16	1.73	0.12	0.05	0.17	0.21	0.05	0.05	0.05	0.23	0.14	0.07	0.02	0.09	0.14	0.05	0.45	0.09	0.88
	R	0.21	0.13	0.03	1.73	0.11	0.07	0.14	0.18	0.01	0.06	0.05	0.09	0.08	0.06	0.03	0.03	0.14	0.29	0.19	0.04	1.18
4	L	0.16	0.06	0.05	0.98	0.24	0.06	0.19	0.32	0.04	0.02	0.05	0.11	0.12	0.04	0.03	0.10	0.04	0.07	0.19	0.08	0.99
	R	0.44	0.25	0.16	1.73	0.08	0.04	0.08	0.18	0.05	0.04	0.07	0.16	0.25	0.05	0.02	0.02	0.16	0.24	0.38	0.21	0.89
5	L	0.32	0.09	0.23	0.92	0.19	0.13	0.43	0.22	0.18	0.06	0.26	0.17	0.47	0.17	0.02	0.18	0.08	0.10	0.34	0.07	0.91
	R	0.40	0.12	0.17	1.54	0.17	0.12	0.30	0.45	0.18	0.03	0.24	0.18	0.05	0.10	0.04	0.06	0.14	0.10	0.25	0.23	0.90
6	L	0.53	0.34	0.08	1.05	0.26	0.15	0.16	0.49	0.04	0.19	0.34	0.19	0.25	0.20	0.10	0.23	0.24	0.31	0.27	0.31	1.00
	R	0.25	0.25	0.23	1.73	0.40	0.03	0.21	0.13	0.09	0.12	0.23	0.23	0.16	0.13	0.09	0.22	0.25	0.28	0.40	0.31	1.73
7	L	0.50	0.29	0.35	1.22	0.47	0.03	0.18	0.52	0.11	0.20	0.27	0.14	0.26	0.25	0.18	0.39	0.21	0.28	0.71	0.28	1.73
	R	0.24	0.10	0.06	1.73	0.17	0.04	0.10	0.23	0.03	0.03	0.14	0.42	0.14	0.08	0.02	0.13	0.04	0.16	0.44	0.09	0.92
8	L	0.67	0.33	0.28	0.88	0.22	0.08	0.25	0.22	0.04	0.08	0.18	0.15	0.20	0.09	0.09	0.19	0.16	0.22	0.59	0.22	1.73
	R	0.27	0.17	0.39	1.73	0.19	0.03	0.16	0.10	0.07	0.14	0.10	0.35	0.37	0.04	0.02	0.08	0.33	0.60	0.45	0.28	1.06
9	L	0.18	0.11	0.29	0.92	0.12	0.19	0.14	0.17	0.01	0.02	0.26	0.22	0.31	0.09	0.03	0.08	0.13	0.12	0.25	0.15	1.73
	R	0.78	0.54	0.52	1.73	0.11	0.12	0.11	0.61	0.14	0.10	0.19	0.03	0.71	0.10	0.15	0.25	0.40	0.45	0.54	0.58	1.21
10	L	0.54	0.13	0.22	1.73	0.17	0.17	0.23	0.10	0.08	0.04	0.20	0.27	0.25	0.08	0.04	0.09	0.14	0.12	0.23	0.19	1.00
	R	0.32	0.08	0.12	1.13	0.13	0.19	0.20	0.11	0.08	0.07	0.26	0.05	0.19	0.06	0.16	0.21	0.14	0.41	0.19	0.09	0.89
11	L	0.30	0.24	0.23	1.00	0.10	0.08	0.13	0.37	0.02	0.01	0.06	0.28	0.23	0.04	0.05	0.06	0.10	0.33	0.27	0.09	0.91
	R	0.56	0.05	0.48	0.94	0.18	0.13	0.15	0.59	0.22	0.10	0.17	0.29	0.60	0.39	0.03	0.20	0.11	0.26	0.88	0.41	1.73
12	L	0.56	0.27	0.22	1.73	0.09	0.11	0.07	0.24	0.10	0.04	0.09	0.18	0.36	0.00	0.06	0.05	0.10	0.07	0.27	0.44	1.73
	R	1.08	0.54	0.62	0.75	0.07	0.25	0.24	0.89	0.20	0.29	0.20	0.12	1.09	0.01	0.19	0.18	0.46	0.23	0.32	0.67	1.73
mean		0.45	0.25	0.25	1.36	0.18	0.11	0.19	0.35	0.10	0.09	0.17	0.22	0.34	0.12	0.08	0.14	0.17	0.22	0.41	0.26	1.29
SD		0.31	0.22	0.16	0.38	0.10	0.07	0.09	0.24	0.09	0.07	0.09	0.16	0.28	0.11	0.09	0.10	0.10	0.13	0.20	0.19	0.38
CV		0.70	0.90	0.67	0.28	0.57	0.61	0.48	0.68	0.86	0.81	0.51	0.71	0.83	0.95	1.02	0.67	0.60	0.60	0.49	0.72	0.30

Subject	Eye	Diacyl-Phosphatidylcholins																
		**PCaaC381**	**PCaaC383**	**PCaaC384**	**PCaaC385**	**PCaaC386**	**PCaaC401**	**PCaaC402**	**PCaaC403**	**PCaaC404**	**PCaaC405**	**PCaaC406**	**PCaaC420**	**PCaaC421**	**PCaaC422**	**PCaaC424**	**PCaaC425**	**PCaaC426**
1	L	0.17	0.31	0.15	0.12	1.52	1.44	0.78	0.42	0.49	0.81	1.44	1.19	1.46	0.87	1.13	1.51	1.35
	R	0.07	0.01	0.03	0.11	0.15	0.27	0.18	0.07	0.07	0.08	0.19	0.09	0.05	0.06	0.06	0.12	0.06
2	L	0.43	0.26	0.21	0.27	0.60	0.19	0.18	0.24	0.28	0.21	0.38	0.40	0.48	0.80	0.09	0.39	0.56
	R	0.15	0.15	0.11	0.16	0.15	0.16	0.58	0.41	0.34	0.04	0.04	0.32	0.35	0.26	0.17	0.12	0.04
3	L	0.06	0.16	0.20	0.16	0.27	0.18	0.28	0.06	0.21	0.05	0.31	0.16	0.35	0.16	0.25	0.27	0.35
	R	0.06	0.21	0.15	0.21	0.23	0.10	0.10	0.09	0.05	0.13	0.27	0.17	0.20	0.03	0.28	0.21	0.19
4	L	0.05	0.08	0.11	0.11	0.11	0.27	0.18	0.24	0.07	0.08	0.27	0.09	0.08	0.18	0.46	0.11	0.08
	R	0.09	0.22	0.17	0.17	0.47	0.42	0.17	0.03	0.19	0.07	0.34	0.45	0.46	0.25	0.42	0.41	0.29
5	L	0.10	0.07	0.08	0.11	0.34	0.47	0.25	0.17	0.31	0.04	0.14	0.18	0.42	0.34	0.06	0.43	0.17
	R	0.16	0.11	0.12	0.22	0.50	0.37	0.08	0.12	0.23	0.11	0.31	0.47	0.37	0.58	0.22	0.26	0.22

6	L	0.17	0.17	0.13	0.23	0.46	0.65	0.16	0.23	0.15	0.19	0.72	0.32	0.45	0.23	0.50	0.57	0.44
	R	0.06	0.22	0.18	0.05	0.15	0.19	0.10	0.17	0.25	0.21	0.27	0.09	0.21	0.41	0.07	0.19	0.22
7	L	0.08	0.19	0.25	0.09	0.55	0.32	0.38	0.09	0.12	0.18	0.35	0.35	0.55	0.31	0.65	0.48	0.25
	R	0.03	0.05	0.02	0.10	0.05	0.19	0.05	0.07	0.07	0.10	0.21	0.14	0.31	0.52	0.20	0.15	0.03
8	L	0.08	0.13	0.20	0.11	0.55	0.37	0.10	0.11	0.23	0.21	0.46	0.35	0.43	0.30	0.13	0.68	0.32
	R	0.07	0.29	0.19	0.43	0.36	0.49	0.34	0.16	0.52	0.19	0.29	0.18	0.27	0.13	0.06	0.17	0.24
9	L	0.11	0.16	0.16	0.07	0.16	0.14	0.20	0.17	0.26	0.08	0.42	0.34	0.12	0.26	0.54	0.22	0.22
	R	0.23	0.37	0.44	0.38	0.88	0.61	0.67	0.48	0.77	0.37	0.79	1.09	0.87	1.09	0.28	0.87	0.80
10	L	0.06	0.20	0.14	0.17	0.58	0.42	0.21	0.22	0.12	0.17	0.34	0.30	0.22	0.50	0.25	0.40	0.18
	R	0.09	0.04	0.08	0.27	0.17	0.11	0.50	0.03	0.05	0.12	0.33	0.28	0.38	0.51	0.14	0.27	0.24
11	L	0.13	0.14	0.18	0.16	0.37	0.13	0.19	0.04	0.26	0.14	0.48	0.54	0.22	0.64	0.47	0.40	0.22
	R	0.11	0.17	0.23	0.21	0.56	0.41	0.41	0.15	0.21	0.23	0.50	0.27	0.50	0.68	0.67	0.58	0.26
12	L	0.02	0.15	0.16	0.11	0.53	0.45	0.20	0.20	0.16	0.13	0.32	0.43	0.37	0.24	0.42	0.59	0.54
	R	0.29	0.47	0.41	0.32	1.10	0.99	0.19	0.02	0.55	0.41	0.92	0.93	1.08	1.05	0.42	1.18	1.01
mean		0.12	0.18	0.17	0.18	0.45	0.39	0.27	0.17	0.25	0.18	0.42	0.38	0.43	0.43	0.33	0.44	0.34
SD		0.09	0.10	0.09	0.09	0.33	0.30	0.19	0.12	0.18	0.16	0.29	0.29	0.31	0.29	0.25	0.34	0.31
CV		0.74	0.57	0.56	0.52	0.73	0.76	0.70	0.74	0.71	0.89	0.69	0.76	0.73	0.67	0.75	0.77	0.90

Subject	Eye	Acyl-Alkyl-Phospatidylcholins																				
		**PCaeC300**	**PCaeC301**	**PCaeC302**	**PCaeC321**	**PCaeC322**	**PCaeC340**	**PCaeC341**	**PCaeC342**	**PCaeC343**	**PCaeC360**	**PCaeC361**	**PCaeC362**	**PCaeC363**	**PCaeC364**	**PCaeC365**	**PCaeC380**	**PCaeC381**	**PCaeC382**	**PCaeC383**	**PCaeC384**	**PCaeC385**
1	L	1.73	1.10	0.53	1.31	0.35	0.39	0.40	0.20	0.95	0.21	0.24	0.60	0.12	0.18	1.25	0.38	0.16	0.12	0.02	0.13	0.26
	R	0.77	0.27	0.11	0.06	0.09	0.12	0.04	0.15	0.16	0.09	0.10	0.14	0.17	0.01	0.08	0.06	0.08	0.17	0.07	0.03	0.09
2	L	0.91	0.31	0.21	0.43	0.23	0.08	0.46	0.21	0.32	0.11	0.19	0.36	0.28	0.10	0.42	0.96	0.43	0.19	0.28	0.32	0.87
	R	0.87	0.29	0.30	0.06	0.11	0.04	0.28	0.40	0.26	0.08	0.02	0.14	0.11	0.17	0.14	0.18	0.42	0.17	0.28	0.20	0.38
3	L	0.24	0.31	0.01	0.38	0.09	0.07	0.09	0.05	0.11	0.10	0.09	0.17	0.18	0.19	0.19	0.28	0.15	0.02	0.24	0.12	0.15
	R	0.19	0.18	0.29	0.18	0.08	0.27	0.25	0.07	0.10	0.13	0.27	0.42	0.15	0.07	0.17	0.33	0.33	0.18	0.17	0.07	0.09
4	L	0.47	0.30	0.15	0.14	0.26	0.21	0.13	0.10	0.08	0.07	0.12	0.18	0.14	0.09	0.01	0.41	0.39	0.14	0.19	0.13	0.10
	R	0.40	0.45	0.15	0.35	0.06	0.06	0.09	0.05	0.30	0.09	0.06	0.15	0.12	0.08	0.28	0.46	0.25	0.13	0.19	0.18	0.19
5	L	0.66	0.24	0.30	0.38	0.14	0.36	0.36	0.25	0.14	0.22	0.41	0.52	0.22	0.04	0.25	0.70	0.62	0.62	0.24	0.20	0.17
	R	1.11	0.33	0.23	0.35	0.25	0.23	0.32	0.34	0.14	0.19	0.31	0.44	0.36	0.18	0.17	0.79	0.71	0.55	0.33	0.29	0.23
6	L	0.49	0.11	0.34	0.35	0.45	0.39	0.31	0.16	0.38	0.40	0.37	0.37	0.30	0.14	0.26	0.69	0.53	0.40	0.33	0.23	0.11
	R	1.06	0.05	0.07	0.20	0.20	0.09	0.07	0.04	0.28	0.07	0.17	0.24	0.24	0.04	0.17	0.91	0.24	0.20	0.16	0.23	0.15
7	L	0.67	0.46	0.58	0.30	0.37	0.34	0.40	0.42	0.32	0.21	0.26	0.29	0.24	0.31	0.30	0.78	0.07	0.07	0.25	0.30	0.14
	R	0.42	0.04	0.20	0.30	0.13	0.22	0.13	0.09	0.10	0.07	0.19	0.21	0.09	0.06	0.14	0.51	0.24	0.20	0.08	0.09	0.15
8	L	0.74	0.44	0.14	0.52	0.18	0.27	0.24	0.08	0.31	0.25	0.34	0.33	0.13	0.11	0.27	0.38	0.36	0.42	0.25	0.09	0.11
	R	0.89	0.23	0.10	0.23	0.14	0.32	0.22	0.07	0.15	0.26	0.30	0.37	0.25	0.26	0.20	0.37	0.50	0.45	0.18	0.24	0.38
9	L	0.45	0.40	0.17	0.34	0.15	0.19	0.21	0.08	0.07	0.19	0.15	0.17	0.11	0.12	0.30	0.76	0.43	0.16	0.12	0.11	0.24
	R	0.87	0.42	0.21	0.68	0.30	0.11	0.23	0.05	0.66	0.34	0.16	0.09	0.31	0.02	0.80	0.95	0.88	0.18	0.12	0.06	0.33
10	L	0.73	0.24	0.29	0.27	0.20	0.23	0.35	0.32	0.25	0.01	0.19	0.30	0.06	0.16	0.28	0.21	0.33	0.13	0.03	0.09	0.19
	R	1.73	0.16	0.25	0.36	0.11	0.22	0.25	0.05	0.17	0.22	0.38	0.44	0.19	0.14	0.09	0.51	0.42	0.18	0.20	0.22	0.12
11	L	0.29	0.37	0.20	0.35	0.07	0.06	0.12	0.08	0.13	0.03	0.04	0.08	0.26	0.16	0.33	0.51	0.19	0.12	0.24	0.17	0.23
	R	0.95	0.51	0.46	0.34	0.31	0.49	0.36	0.39	0.34	0.33	0.40	0.47	0.35	0.44	0.44	0.68	0.16	0.38	0.18	0.18	0.19
12	L	1.33	0.40	0.31	0.52	0.05	0.08	0.15	0.16	0.30	0.14	0.08	0.19	0.19	0.07	0.50	0.83	0.28	0.09	0.07	0.16	0.20
	R	1.50	0.96	0.65	0.87	0.32	0.12	0.37	0.39	0.90	0.34	0.11	0.36	0.21	0.23	0.97	0.68	0.16	0.12	0.11	0.17	0.33
mean		0.81	0.36	0.26	0.39	0.19	0.21	0.24	0.18	0.29	0.17	0.21	0.29	0.20	0.14	0.33	0.56	0.35	0.22	0.18	0.17	0.23
SD		0.43	0.24	0.16	0.26	0.11	0.12	0.12	0.13	0.23	0.10	0.12	0.14	0.08	0.10	0.29	0.25	0.20	0.15	0.09	0.08	0.16
CV		0.52	0.67	0.60	0.67	0.57	0.60	0.49	0.75	0.80	0.61	0.57	0.48	0.41	0.69	0.86	0.45	0.56	0.69	0.48	0.46	0.71

Subject	Eye	Acyl-Alkyl-Phospatidylcholins																
		**PCaeC386**	**PCaeC401**	**PCaeC402**	**PCaeC403**	**PCaeC404**	**PCaeC405**	**PCaeC406**	**PCaeC420**	**PCaeC421**	**PCaeC422**	**PCaeC423**	**PCaeC424**	**PCaeC425**	**PCaeC443**	**PCaeC444**	**PCaeC445**	**PCaeC446**
1	L	0.37	0.20	0.40	1.12	0.08	0.46	1.50	1.46	0.55	0.34	0.89	1.51	1.21	1.47	1.18	1.25	0.66
	R	0.21	0.12	0.14	0.14	0.09	0.17	0.09	0.03	0.10	0.17	0.89	0.13	0.14	0.10	0.14	0.19	0.04
2	L	0.87	0.32	0.34	0.29	0.45	0.21	0.49	0.49	0.76	0.58	1.00	0.42	0.04	0.43	0.57	0.35	0.35
	R	0.58	0.41	0.22	0.06	0.10	0.10	0.13	0.04	0.39	0.20	1.00	0.10	0.18	0.07	0.18	0.12	0.11
3	L	0.22	0.04	0.16	0.06	0.11	0.12	0.22	0.13	0.17	0.11	0.45	0.22	0.06	0.30	0.19	0.17	0.02
	R	0.13	0.20	0.43	0.20	0.26	0.09	0.21	0.15	0.40	0.48	0.42	0.18	0.41	0.20	0.39	0.15	0.04
4	L	0.08	0.22	0.31	0.23	0.12	0.15	0.13	0.16	0.33	0.43	0.81	0.17	0.16	0.25	0.26	0.24	0.04
	R	0.17	0.07	0.16	0.25	0.12	0.09	0.48	0.42	0.20	0.04	1.73	0.37	0.43	0.40	0.16	0.25	0.09
5	L	0.69	0.47	0.79	0.36	0.37	0.11	0.44	0.24	0.64	0.63	1.39	0.29	0.13	0.41	0.34	0.18	0.06
	R	0.25	0.36	0.56	0.29	0.28	0.22	0.32	0.36	0.47	0.44	1.73	0.35	0.43	0.38	0.35	0.33	0.02
6	L	0.11	0.46	0.41	0.39	0.39	0.18	0.60	0.48	0.56	0.58	0.94	0.57	0.55	0.52	0.55	0.36	0.03
	R	0.16	0.16	0.04	0.03	0.08	0.14	0.28	0.24	0.17	0.15	1.01	0.17	0.38	0.24	0.06	0.14	0.09
7	L	0.48	0.35	0.10	0.21	0.20	0.38	0.49	0.37	0.39	0.30	1.73	0.42	0.51	0.38	0.09	0.41	0.14
	R	0.15	0.11	0.34	0.12	0.21	0.07	0.15	0.10	0.40	0.40	1.73	0.13	0.28	0.17	0.20	0.14	0.05
8	L	0.42	0.34	0.44	0.29	0.23	0.17	0.63	0.61	0.52	0.55	1.04	0.59	0.38	0.51	0.13	0.20	0.02
	R	0.35	0.49	0.72	0.21	0.24	0.10	0.27	0.35	0.80	0.68	1.73	0.24	0.27	0.13	0.16	0.05	0.03
9	L	0.47	0.36	0.23	0.18	0.08	0.06	0.16	0.09	0.21	0.34	1.73	0.08	0.42	0.19	0.07	0.05	0.07
	R	0.94	0.07	0.39	0.60	0.10	0.05	0.81	0.80	0.28	0.17	1.00	0.85	0.84	0.88	0.70	0.28	0.42

10	L	0.32	0.16	0.29	0.23	0.13	0.10	0.48	0.40	0.68	0.58	1.73	0.44	0.24	0.26	0.12	0.19	0.06
	R	0.35	0.25	0.48	0.38	0.32	0.11	0.29	0.24	0.34	0.61	1.12	0.18	0.29	0.29	0.41	0.28	0.10
11	L	0.31	0.08	0.16	0.23	0.28	0.04	0.27	0.27	0.25	0.14	1.00	0.26	0.42	0.27	0.31	0.20	0.12
	R	0.34	0.21	0.55	0.10	0.54	0.32	0.54	0.58	0.63	0.70	1.73	0.49	0.46	0.42	0.08	0.15	0.14
12	L	0.46	0.28	0.31	0.35	0.24	0.02	0.64	0.46	0.42	0.27	1.00	0.62	0.55	0.66	0.43	0.57	0.09
	R	0.49	0.40	0.13	0.70	0.06	0.36	1.06	1.03	0.81	0.78	1.00	0.97	1.11	1.01	1.01	0.92	0.16
mean		0.37	0.26	0.34	0.29	0.21	0.16	0.45	0.40	0.43	0.40	1.20	0.41	0.41	0.41	0.34	0.30	0.12
SD		0.22	0.14	0.19	0.23	0.13	0.11	0.32	0.32	0.20	0.21	0.42	0.32	0.29	0.31	0.29	0.27	0.15
CV		0.59	0.53	0.56	0.79	0.60	0.71	0.72	0.82	0.47	0.53	0.35	0.80	0.70	0.76	0.85	0.90	1.19

Subject	Eye	Sphingomyelins														
		**SMOHC141**	**SMOHC161**	**SMOHC221**	**SMOHC222**	**SMOHC241**	**SMC160**	**SMC161**	**SMC180**	**SMC181**	**SMC202**	**SMC223**	**SMC240**	**SMC241**	**SMC260**	**SMC261**
1	L	0.03	0.11	0.06	0.21	0.05	0.41	0.02	0.03	0.56	1.57	0.10	0.15	0.18	0.42	0.73
	R	0.06	0.09	0.02	0.00	0.02	0.06	0.07	0.13	0.65	0.97	0.07	0.02	0.20	0.21	0.07
2	L	0.44	0.16	0.30	0.17	0.05	0.37	0.17	0.20	1.32	0.26	0.05	0.08	0.24	0.25	0.66
	R	0.22	0.15	0.16	0.14	0.01	0.16	0.06	0.18	1.73	0.50	0.14	0.03	0.24	0.37	0.17
3	L	0.06	0.15	0.20	0.14	0.01	0.09	0.12	0.12	0.32	0.69	0.06	0.04	0.34	0.17	0.50
	R	0.10	0.09	0.07	0.06	0.01	0.05	0.16	0.15	0.32	0.35	0.09	0.08	0.20	0.15	0.25
4	L	0.05	0.03	0.02	0.06	0.02	0.14	0.03	0.23	0.05	1.24	0.04	0.05	0.17	0.08	0.17
	R	0.02	0.14	0.23	0.07	0.02	0.11	0.02	0.18	0.88	0.31	0.02	0.07	0.22	0.05	0.63
5	L	0.14	0.21	0.19	0.20	0.08	0.18	0.09	0.26	0.37	1.00	0.17	0.20	0.08	0.17	0.08
	R	0.05	0.16	0.19	0.22	0.06	0.24	0.07	0.35	0.30	0.48	0.14	0.14	0.20	0.33	0.13
6	L	0.04	0.09	0.13	0.13	0.03	0.08	0.08	0.17	0.92	0.87	0.09	0.15	0.14	0.18	0.35
	R	0.06	0.09	0.16	0.22	0.03	0.23	0.07	0.23	0.58	0.37	0.06	0.10	0.26	0.31	0.13
7	L	0.08	0.06	0.49	0.05	0.02	0.22	0.02	0.41	0.83	0.63	0.06	0.06	0.29	0.12	0.57
	R	0.17	0.05	0.09	0.02	0.01	0.05	0.09	0.13	0.59	0.34	0.05	0.10	0.33	0.15	0.19
8	L	0.07	0.15	0.18	0.19	0.00	0.08	0.06	0.17	0.25	0.97	0.05	0.10	0.28	0.17	0.58
	R	0.05	0.09	0.27	0.25	0.01	0.16	0.11	0.30	0.80	0.26	0.11	0.15	0.14	0.08	0.73
9	L	0.08	0.17	0.35	0.05	0.02	0.19	0.07	0.19	0.93	0.54	0.04	0.08	0.23	0.20	0.37
	R	0.21	0.23	0.28	0.23	0.03	0.10	0.08	0.44	0.26	0.67	0.03	0.09	0.33	0.32	0.35
10	L	0.06	0.14	0.25	0.20	0.02	0.12	0.11	0.21	0.56	0.71	0.09	0.02	0.56	0.49	0.32
	R	0.08	0.16	0.21	0.07	0.05	0.15	0.06	0.32	0.52	0.92	0.11	0.21	0.06	0.24	0.30
11	L	0.04	0.08	0.25	0.07	0.01	0.11	0.04	0.11	0.44	0.87	0.07	0.04	0.17	0.13	0.33
	R	0.12	0.06	0.13	0.02	0.05	0.14	0.07	0.13	0.87	0.79	0.15	0.06	0.27	0.25	0.54
12	L	0.15	0.04	0.07	0.08	0.06	0.08	0.11	0.18	0.45	0.64	0.15	0.09	0.24	0.32	0.51
	R	0.16	0.19	0.16	0.27	0.03	0.05	0.08	0.38	0.27	0.74	0.14	0.17	0.63	0.26	0.39
mean		0.11	0.12	0.19	0.13	0.03	0.15	0.08	0.22	0.62	0.70	0.09	0.09	0.25	0.23	0.38
SD		0.09	0.05	0.11	0.08	0.02	0.09	0.04	0.10	0.37	0.32	0.04	0.05	0.13	0.11	0.20
CV		0.83	0.45	0.58	0.62	0.70	0.62	0.50	0.46	0.60	0.46	0.49	0.56	0.50	0.48	0.54
