# Supplementary material for: Combined Targeted Analysis of Metabolites and Proteins in Tear Fluid With Regard to Clinical Applications
Source: Transl Vis Sci Technol. 2018 Dec 6;7(6):22. doi: 10.1167/tvst.7.6.22 (PMC6284467; doi:10.1167/tvst.7.6.22)

**Title:** Combined Targeted Analysis of Metabolites and Proteins in Tear Fluid with Regard to Clinical Applications
**Jounal:** TVST
**Authors:** Sascha Dammeier, Peter Martus, Franziska Klose, Michael Seid, Dario Bosch, Janina D'Alvise, Focke Ziemssen, Spyridon Dimopoulos and Marius Ueffing
**Corresponding Author:** Sascha Dammeier, Institute for Ophthalmic Research, Core Facility for Medical Bioanalytics, University of Tübingen, Elfriede-Aulhorn-Strasse 7, 72076 Tübingen, Germany, email: sascha.dammeier@uni-tuebingen.de

**Supplementary Figure S1.** Relative analyte concentrations in tear fluid of three different subjects considering the individual eyes. Data of subject 1 (mean tear secretion rates: left 1.33±0.19 mm/min, right 2.18±0.14 mm/min), subject 2 (mean tear secretion rates: left 56.67±6.60 mm/min, right 68.67±15.52 mm/min), and subject 3 (mean tear secretion rates: left 3.67±0.25 mm/min, right 3.67±0.25 mm/min) are shown for (A) amino acids, (B) acyl carnitines, (C) proteins, (D) lyso-phosphatidylcholines, (E) diacyl-phosphatidylcholines, and (F) sphingomyelins at three different visits

**A**

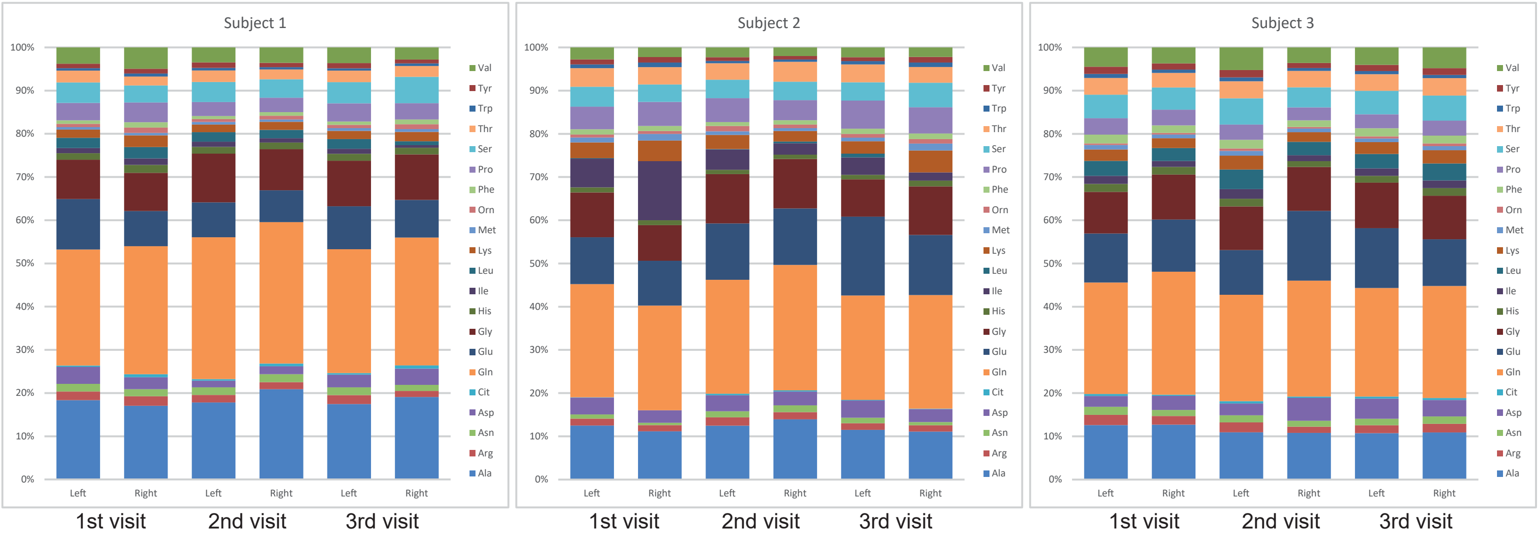


**B**

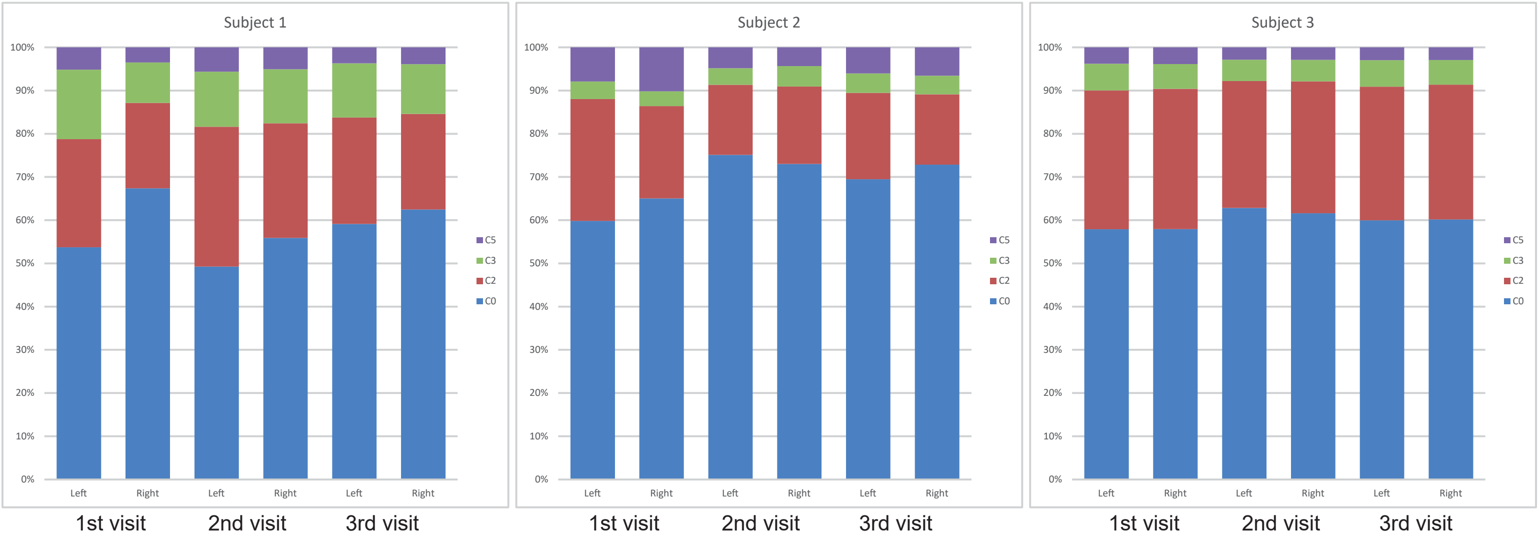


**C**

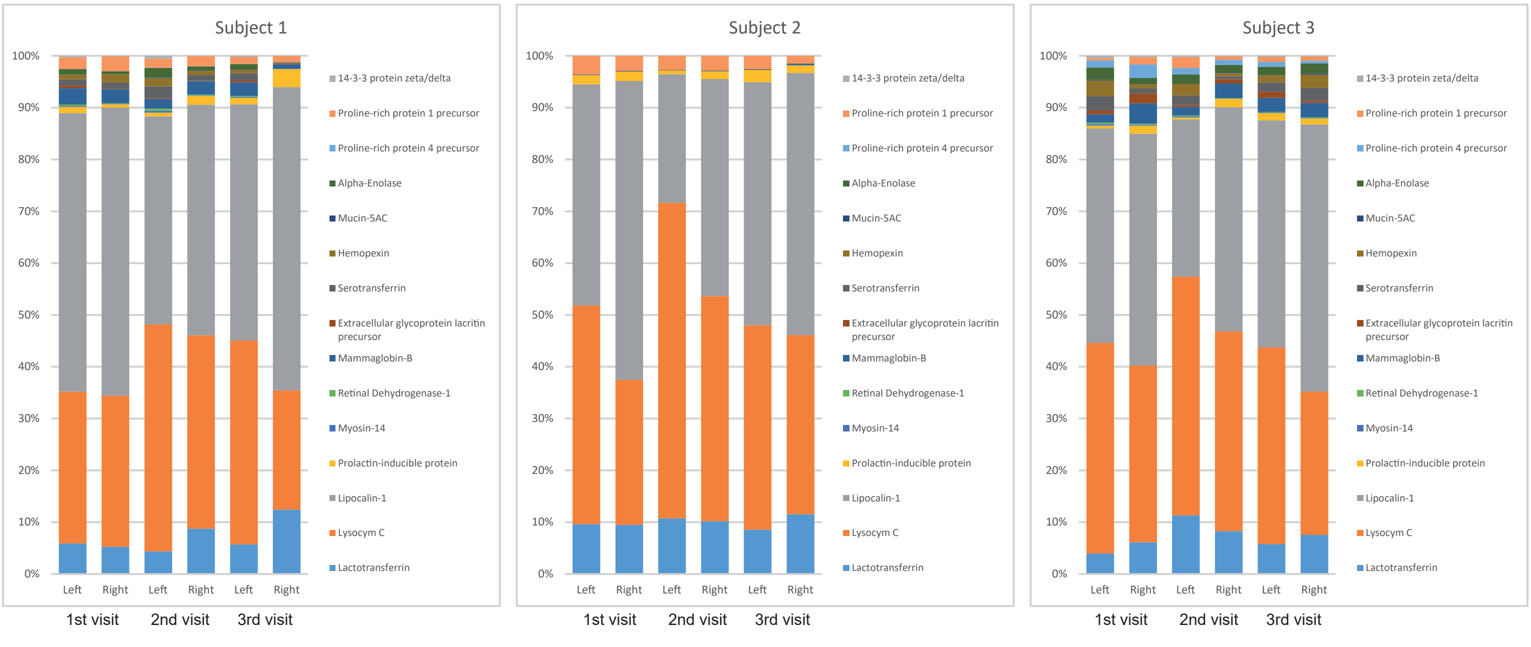

**Supplementary Figure S1.** continued

## D

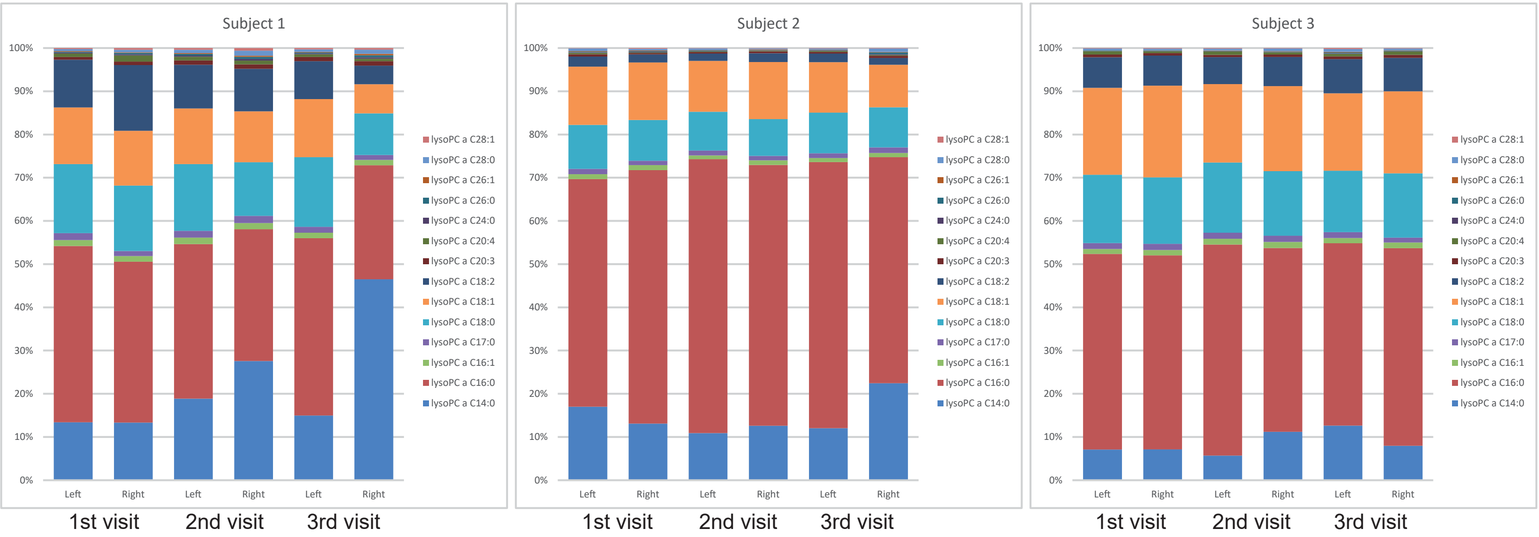


## E

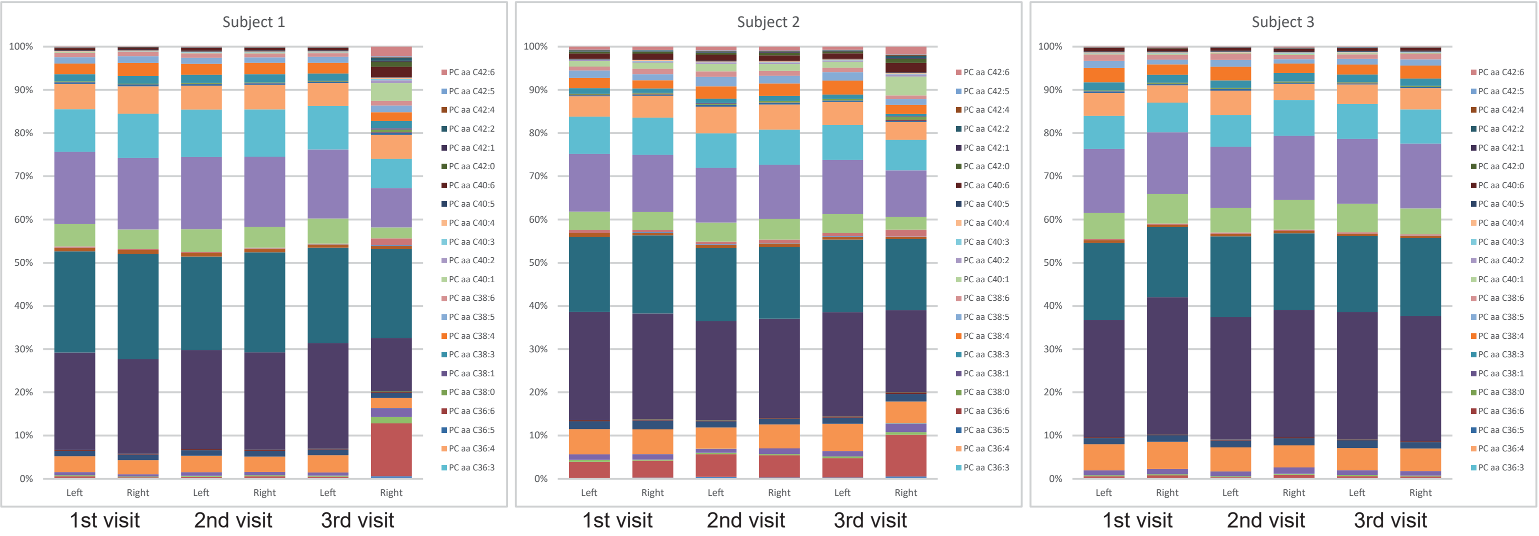


## F

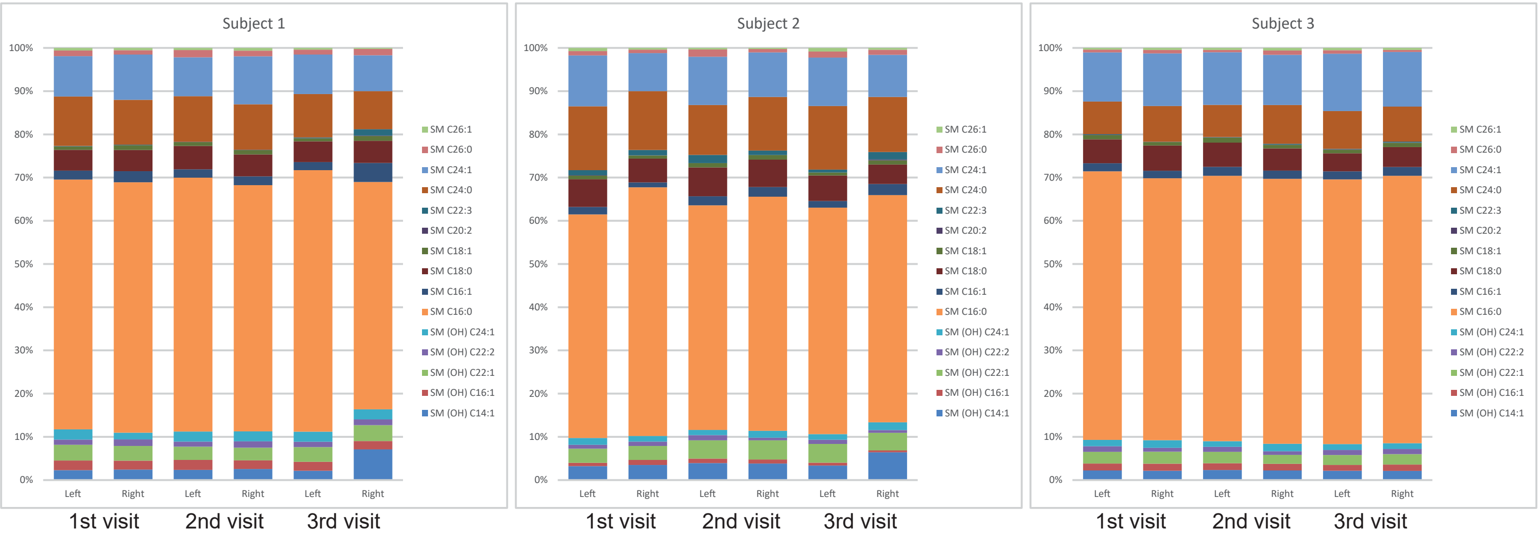

Supplement: Supplement 5 [file tvst-07-06-18_s05.pdf]
