# Supplementary material for: Combined Targeted Analysis of Metabolites and Proteins in Tear Fluid With Regard to Clinical Applications
Source: Transl Vis Sci Technol. 2018 Dec 6;7(6):22. doi: 10.1167/tvst.7.6.22 (PMC6284467; doi:10.1167/tvst.7.6.22)

Title: Combined Targeted Analysis of Metabolites and Proteins in Tear Fluid with Regard to Clinical Applications
Jounal: TVST
Authors: Sascha Dammeier, Peter Martus, Franziska Klose, Michael Seid, Dario Bosch, Janina D'Alvise, Focke Ziemssen, Spyridon Dimopoulos and Marius Ueffing
Corresponding Author: Sascha Dammeier, Institute for Ophthalmic Research, Core Facility for Medical Bioanalytics, University of Tübingen, Elfriede-Aulhorn-Strasse 7, 72076 Tübingen, Germany, email: sascha.dammeier@uni-tuebingen.de

Supplementary Figure S2. Relative concentrations of 15 proteins in tear fluid of individual study subjects determined by targeted proteomics following metabolite extraction (6 donations per subject).

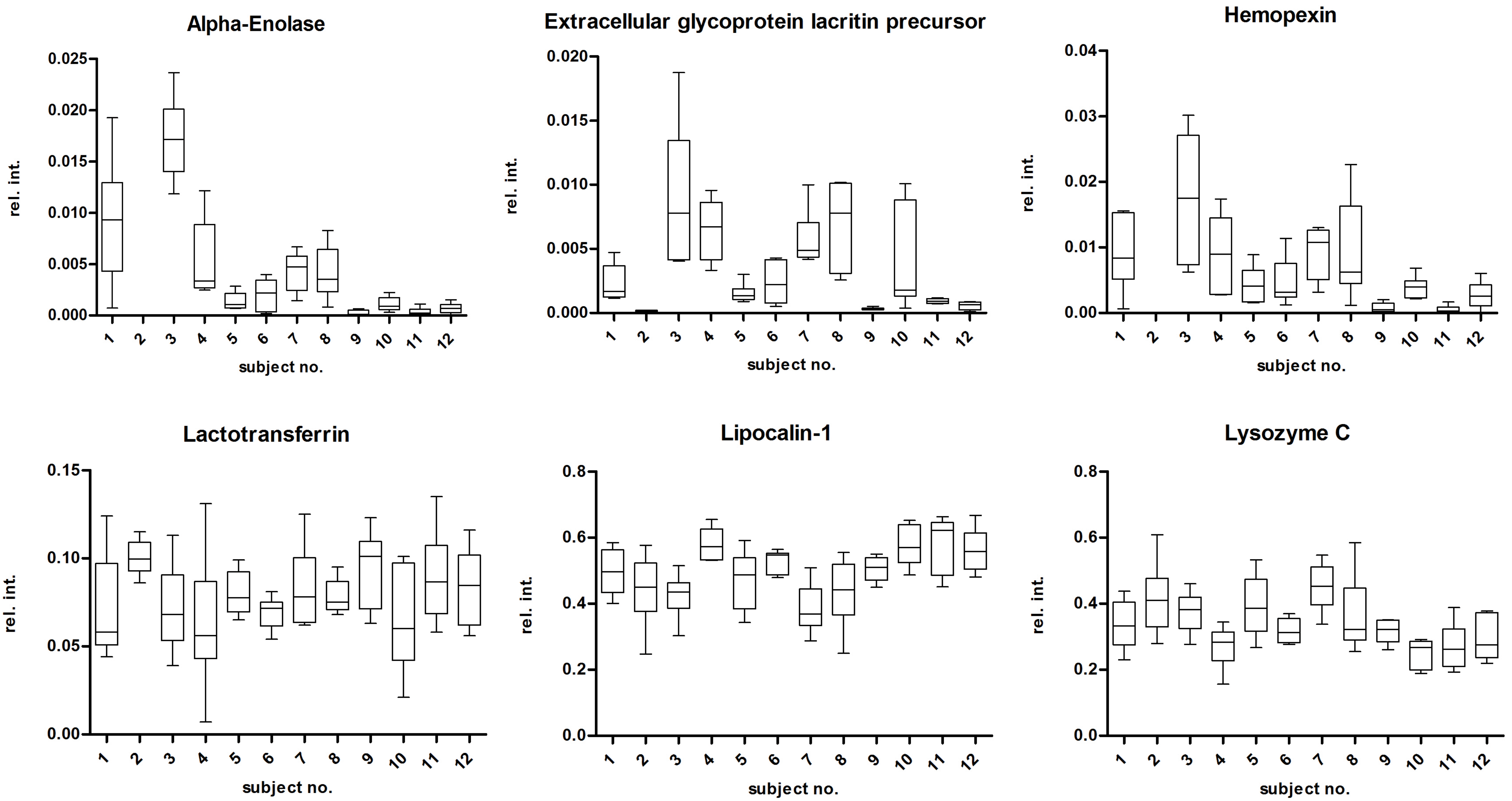

Supplementary Figure S2. continued

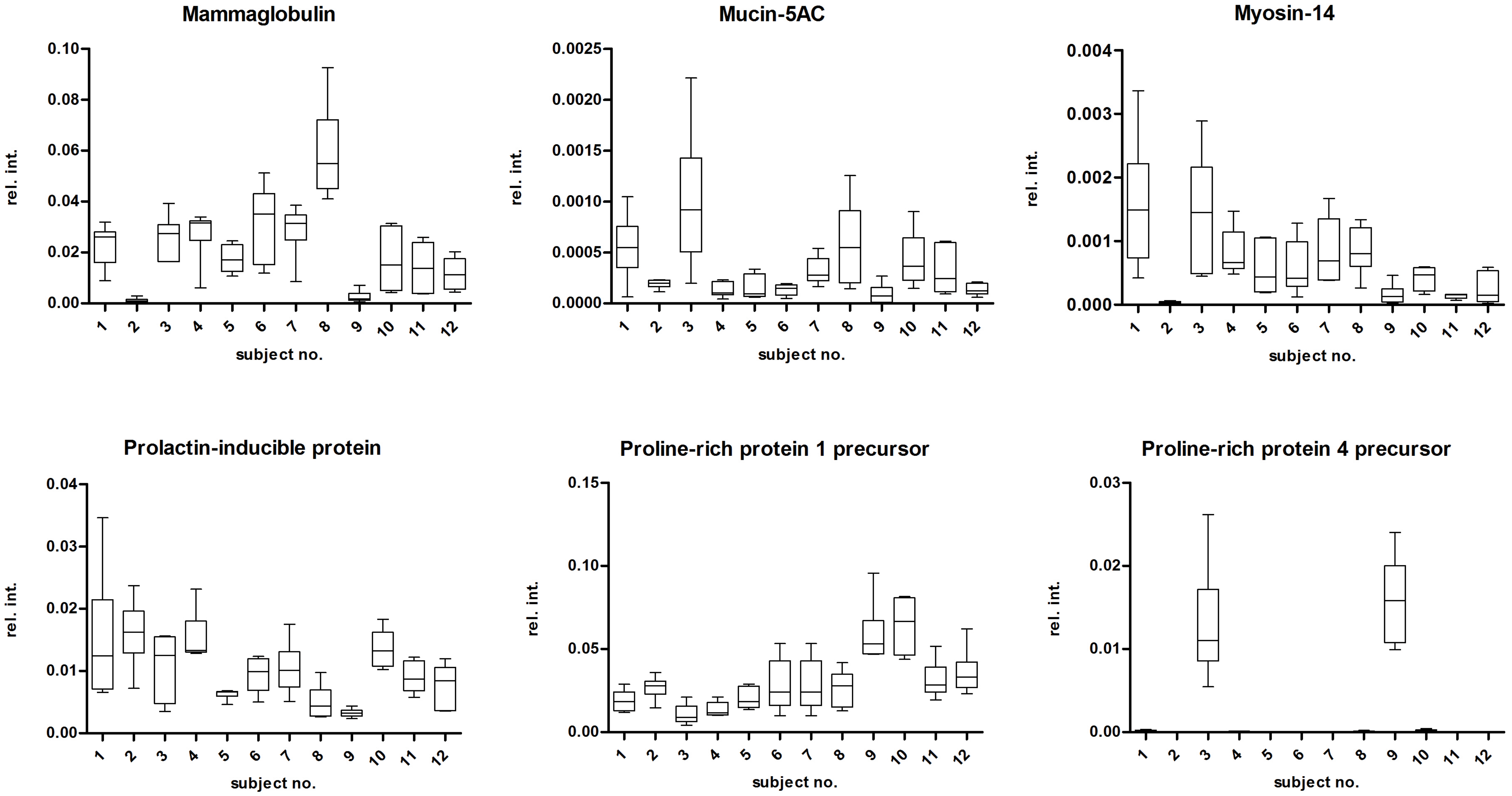

Supplementary Figure S2. continued

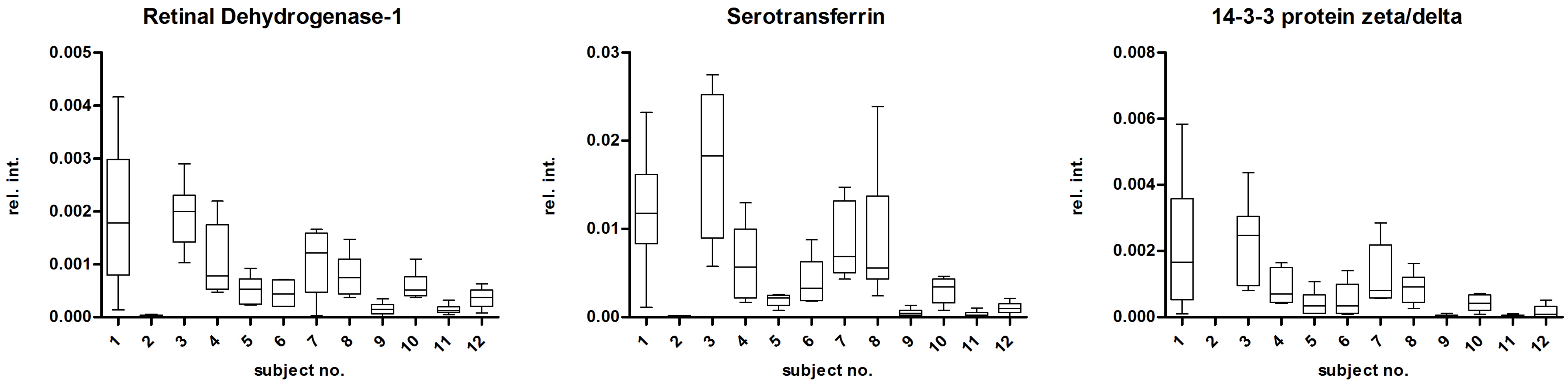

Supplement: Supplement 6 [file tvst-07-06-18_s06.pdf]
