# Supplementary material for: Combined Targeted Analysis of Metabolites and Proteins in Tear Fluid With Regard to Clinical Applications
Source: Transl Vis Sci Technol. 2018 Dec 6;7(6):22. doi: 10.1167/tvst.7.6.22 (PMC6284467; doi:10.1167/tvst.7.6.22)
Supplement: Supplement 7 [file tvst-07-06-18_s07.pdf]

Title: Combined Targeted Analysis of Metabolites and Proteins in Tear Fluid with Regard to Clinical Applications

Jounal: TVST

Authors: Sascha Dammeier, Peter Martus, Franziska Klose, Michael Seid, Dario Bosch, Janina D‘Alvise, Focke Ziemssen, Spyridon Dimopoulos and Marius Ueffing

Corresponding Author:

Sascha Dammeier, Institute for Ophthalmic Research, Core Facility for Medical Bioanalytics, University of Tübingen, Elfriede-Aulhorn-Strasse 7, 72076 Tübingen, Germany, email: sascha.dammeier@uni-tuebingen.de

SUPPLEMENTARY TABLE S5. Calculation of standard errors of relevant coefficients of variation. To approximate the confidence intervals standard errors (SE) have been calculated for the relevant CVs and sample numbers (n = 12, 24, 36).

CV	SE(CV), n=12	SE(CV), n=24	SE(CV), n=36
0.1	0.022	0.015	0.012
0.2	0.044	0.031	0.025
0.3	0.069	0.048	0.039
0.4	0.098	0.068	0.055
0.5	0.131	0.090	0.073
0.6	0.168	0.116	0.094
0.7	0.210	0.145	0.118
0.8	0.258	0.178	0.144
